# A Bench‐Stable Fluorophosphine Nickel(0) Complex and Its Catalytic Application

**DOI:** 10.1002/anie.202506271

**Published:** 2025-04-18

**Authors:** Franziska Flecken, Arjun Neyyathala, Toni Grell, Schirin Hanf

**Affiliations:** ^1^ Department Karlsruhe Institute of Technology Institute for Inorganic Chemistry Engesserstr. 15 76131 Karlsruhe Germany; ^2^ Dipartimento di Chimica Institution Università degli Studi di Milano Via Camillo Golgi 19 Milan 20131 Italy

**Keywords:** Coupling reactions, Fluorophosphines, In situ reduction, Nickel(0), Pre‐catalyst

## Abstract

We herein present a fluorophosphine‐based nickel(0) complex [Ni(PFPh_2_)_4_] (**1**), which is highly stable in air and water. [Ni(PFPh_2_)_4_] can be obtained from a one‐pot reaction of [Ni(MeCN)_4_](BF_4_)_2_ with Ph_2_P(═O)–PPh_2_, involving a unique in situ reduction of Ni(II) to Ni(0) and a simultaneous fluorination by the BF_4_^−^ anion. This complex does not only incorporate a nickel center in the zero‐oxidation state, resulting from a Ni(II) precursor, but also includes fluorophosphine ligands, which typically disproportionate immediately in solution. The application of [Ni(PFPh_2_)_4_] as highly stable Ni(0) pre‐catalyst in combination with additional phosphine ligands, such as dppf (1,1′‐bis(diphenylphosphino)ferrocene), in various coupling reactions uncovers its high catalytic activity and versatility, which is superior to [Ni(COD)_2_] (COD═cycloocta‐1,5‐diene) as conventional Ni(0) source.

## Graphical abstract

We herein present the synthesis of a bench‐stable Ni(0) complex, [Ni(PFPh_2_)_4_], stabilized by vastly underexplored fluorophosphine ligands. The application of [Ni(PFPh_2_)_4_] in Suzuki–Miyaura and Kumada‐Tamao‐Corriu cross‐coupling reactions as well as in Buchwald–Hartwig C─N bond formation reactions, indicates its promising potential as alternative to conventional Ni(0) sources.

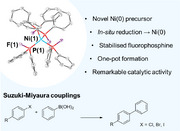

## Introduction

For a long time, palladium‐based catalysts dominated the area of catalytic coupling reactions.^[^ ^1^ ^]^ However, in the last years, nickel catalysts were shown to be capable of substituting or even outperforming palladium‐based systems. This is the result of significant achievements in the tailored design of ligands, which enables modular modifications of the steric and electronic properties of the metal center.^[^ ^2^, ^3^, ^4^, ^5^ ^]^ Contrary to palladium, nickel is cheaper, more abundant, and possesses distinguished properties, such as its smaller atomic radius, lower reduction potential, and electronegativity.^[^ ^3^, ^6^ ^]^ These properties explain why palladium‐based catalytic cycles primarily involve Pd(0) and Pd(II) states, while nickel catalysis includes oxidation states ranging from 0 to +III, thus enabling reaction pathways through one‐electron reduction processes.^[^ ^1^, ^2^, ^3^, ^4^, ^5^, ^7^, ^8^, ^9^ ^]^ Another key difference is that nickel is much more prone to undergo oxidative addition reactions with a substrate, in comparison with its heavier analogues platinum and palladium.^[^ ^2^ ^]^ This increased reactivity allows for the activation of challenging substrates, such as aryl chlorides and alkyl halides.^[^ ^10^, ^11^, ^12^ ^]^ On the downside, the susceptibility of Ni(0) toward oxidative addition reactions also implies that Ni(0) compounds, which are the starting point of most nickel‐catalyzed reactions, are easily oxidized by oxidizing agents, such as air.^[^ ^13^ ^]^ To address this difficulty, more stable Ni(II) complexes have been applied as catalyst precursors in many reactions, which will then be reduced in situ through the use of strong reducing agents to obtain the catalytically active Ni(0) species.^[^ ^14^, ^15^ ^]^

Alternatively, Ni(0) compounds can be used directly. In this context, [Ni(COD)_2_] (COD═cycloocta‐1,5‐diene) dominates the field, despite its high price and its air‐, moisture‐, light‐, and heat‐sensitivity. Notwithstanding the versatile use of [Ni(COD)_2_] as a catalyst precursor, the COD ligand itself, which is released from the initial complex during the catalytic reaction, is known to block active sites, resulting in catalyst poisoning.^[^ ^13^, ^16^, ^17^, ^18^, ^19^ ^]^ Therefore, there is a need for the development of alternative, cheaper, and easier‐to‐handle nickel(0) sources.^[^ ^14^, ^20^, ^21^ ^]^ Important steps toward this direction were achieved by Cornella and co‐workers, who synthesized a highly stable *p*‐CF_3_‐stilben‐based Ni(0)–olefin complex^[^ ^14^, ^20^, ^21^ ^]^ and Engle et al. who discovered [Ni(COD)(L)] complexes, where L refers to quinone, cyclopentadienone, thiophene‐S‐oxide, or fulvene ligands.^[^ ^16^, ^19^ ^]^

**Figure 1:**
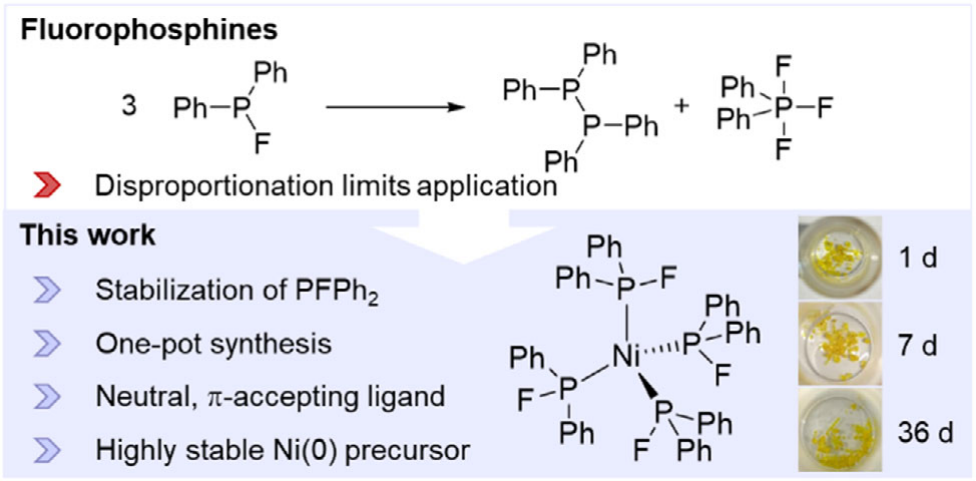
Disproportionation reaction of a fluorophosphines forming P─P coupled products and difluorophosphoranes (top). In this work, a highly stable Ni(0) complex stabilized by fluorophosphine ligands has been synthesized and can be considered as alternative Ni(0) source (bottom). The compound can be stored in solid form over several days on the bench (photographs).

We herein present an alternative to conventional Ni(0) sources, namely [Ni(PFPh_2_)_4_] (**1**), a nickel complex stabilized by monodentate fluorophosphine ligands, which is highly air‐ and moisture‐stable in its solid form for an extended period of time. This Ni(0) complex not only stands out due to its unusually high stability, but also due to the presence of intact fluorophosphines, a ligand class known to be highly unstable due to disproportionation reactions into unfluorinated P─P coupled products and difluorophosphoranes (Figure 1). To date, only a few examples of coordinated fluorophosphines with long‐term stability in solution have been reported, most of which are stabilized by sterically demanding or electron‐withdrawing substituents on the phosphorus centers, such as *^t^* Bu or CF_3_.^[^ ^22^, ^23^, ^24^, ^25^, ^26^, ^27^, ^28^, ^29^, ^30^, ^31^ ^]^ Alternatively, some complexes of less‐stable fluorophosphines, such as PFPh_2_, could be synthesized by direct fluorination of an already coordinated phosphine ligand.^[^ ^32^, ^33^, ^34^, ^35^ ^]^ An overview of literature‐reported fluorophosphines is provided in the ESI (Table S3). Even sparser are the studies concerning the catalytic application of fluorophosphine‐based metal complexes. There is (to the best of our knowledge) only one example of Fey and co‐workers, who describe the application of Ni‐ and Rh‐based catalysts, including electron‐withdrawing and bulky PFR_2_ ligands (R ═ phosphatrioxa‐adamantane or phobanes 9‐phosphabicyclo[3.3.1]nonane), in hydrocyanation and hydroformylation reactions.^[^ ^30^ ^]^

Despite this research gap, fluorophosphine ligands exhibit strong π‐accepting and *σ*‐donating properties, making them promising ligand candidates for stabilizing various metal oxidation states throughout a catalytic cycle.^[^ ^36^ ^]^ Therefore, in this work, we investigate the application of complex **1** as catalyst in coupling reactions.

## Results and Discussion

### Synthesis and Characterization

**Figure 2:**
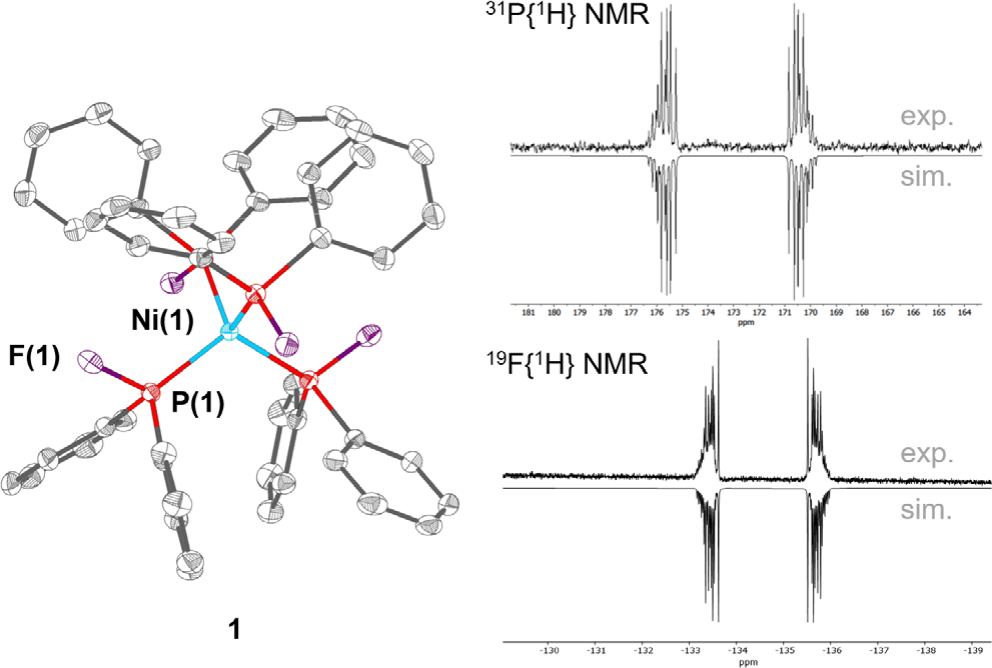
Left side: molecular structure of compound **1**. Hydrogen atoms are emitted for clarity and displacement ellipsoids are drawn at 30% probability. Selected bond lengths (Å) and angles (°): Ni(1)–P(1) 2.1296(4), F(1)–P(1) 1.6465(12), P(1)–C(2) 1.8211(19), P(1)–C(3) 1.8232(19), P(1)–Ni(1)–P(1) 108.732(12)–110.96(2), F(1)–P(1)–Ni(1) 114.97(5), F(1)–P(1)–C(2) 97.42(7), F(1)–P(1)–C(3) 97.17(7), C(2)–P(1)–Ni(1) 117.08(6), C(3)–P(1)–Ni(1) 124.53(6). Right side: experimental (in C_6_D_6_) and simulated ^31^P{^1^H} (173.1 ppm, AA′A″A″′XX′X″X″′, ^2^ *J*_PP_ = −23.26 Hz, ^1^ *J*_PF_ = −850.25 Hz, ^3^ *J*_PF_ = +45.50 Hz) and ^19^F{^1^H} NMR spectra (−134.6 ppm AA′A″A″′XX′X″X″′, ^1^ *J*_PF_ = −850.25 Hz, ^3^ *J*_PF_ = +45.50 Hz, ^4^ *J*_FF_ = −1.4 Hz).

The one‐pot synthesis of [Ni(MeCN)_4_](BF_4_)_2_ with Ph_2_P(═O)–PPh_2_ (PPO) leads to the formation of the Ni(0) compound [Ni(PFPh_2_)_4_] (**1**). Yellow crystals of **1** in 25% crystalline yield can be obtained through layering a saturated solution of dichloromethane (DCM) with acetonitrile. The molecular structure of **1**, determined by single‐crystal X‐ray diffraction (SCXRD), is shown in Figure 2. Within **1**, the Ni(0) atom is tetrahedrally coordinated by four diphenyl fluorophosphine ligands. Compared to the only other reported nickel‐coordinating PFR_2_ ligand, in [NiBr_2_(PF*^t^* Bu_2_)_2_], where an electronically‐stabilized fluorophosphine is coordinated, in **1** the Ni─P bond length is shorter and the P─F bond is longer (**1**: Ni–P 2.1296(4) Å; P–F 1.6465(12) Å and [NiBr_2_(PF*^t^* Bu_2_)_2_]: Ni–P 2.232(3) Å; P–F 1.579(7) Å).^[^ ^28^ ^]^ These observations strongly indicate the unique electronic character of the PFPh_2_ ligand (see ESI, Table S3 for further comparisons).

Further analyses of complex **1** are consistent with the findings from SCXRD. Powder X‐ray diffraction (PXRD, ESI, Figure S46) shows that the bulk material corresponds to the observed crystal structure and is phase pure. The results from mass spectrometry with electrospray ionization (ESI‐MS, ESI, Figures S48–S50) and elemental analysis are in line with the molecular formula. Sharp signals in the ^1^H, ^19^F, and ^31^P NMR spectra (ESI, Figures S1–S4) and the absence of a signal in the EPR spectrum (ESI, Figure S47) confirm the diamagnetic nature of the Ni(0) complex. Furthermore, by using automated line‐shape analysis (see ESI) of the complex, higher‐order multiplets observed in the ^19^F{^1^H} (R = 0.8%) and ^31^P{^1^H} NMR (R = 0.6%) spectra of **1** could in both cases successfully be refined as the expected AA′A′′A′′′XX′X′′X′′′ (*T_d_* symmetrization) spin system. The analysis yielded parameters consistent with the molecular structure involving a multiplet at 173.1 ppm in the ^31^P{^1^H} NMR spectrum as well as a multiplet at −134.6 ppm in the ^19^F{^1^H} NMR spectrum (Figure 2) and indicates a free rotation of the coordinated phosphine ligands around the Ni─P axes.

### Mechanistic Studies

The synthesis of **1** involves a unique in situ reduction and simultaneous fluorination of the phosphine precursor by the BF_4_^−^ anion. To the best of our knowledge, in literature, either F^−^‐induced reductions of metal phosphine complexes based on Pd and Ni have been reported, which do not include a simultaneous fluorination and the subsequent formation of a fluorophosphine ligand,^[^ ^37^, ^38^, ^39^, ^40^, ^41^, ^42^ ^]^ or BF_4_^−^‐mediated fluorinations have been discussed, which however are not accompanied by a simultaneous metal reduction.^[^ ^34^, ^35^ ^]^ Thus, the synthesis of **1** is the first example which combines both processes in one reaction.

**Figure 3:**
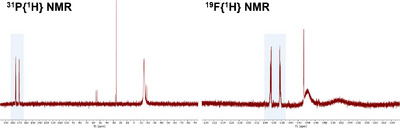
^31^P{^1^H} and ^19^F{^1^H} NMR spectra of the reaction solution of PPO and [Ni(MeCN)_4_](BF_4_)_2_ in DCM (1:4 ratio) after 0.5 h. Signals corresponding to [Ni(PFPh_2_)_4_] are highlighted in blue. The remaining signals can be attributed to intermediates and by‐products of the reaction, whereby no residual starting materials can be observed.

To gain insights into the unusual reduction and fluorination mechanism, detailed NMR spectroscopic investigations of the reaction solutions have been conducted to identify potential intermediates and by‐products (Figure 3). First, the role and the requirement of the individual components of the reaction were investigated. Neither the reaction of PPO with other BF_4_^−^ salts, such as NEt_4_BF_4_ and NH_4_BF_4_, nor the reaction of PPO with other metal precursors, such as [Cu(MeCN)_4_](BF_4_)_2_, result in the formation of any fluorophosphines or similar metal complexes (ESI, Figures S20–S23). This underlines the unique combination of nickel, BF_4_^−^ and the PPO ligand for the reaction to proceed.

The impact of the reaction stoichiometry was investigated using reaction solutions of the starting materials [Ni(MeCN)_4_](BF_4_)_2_ and PPO in various ratios (1:1, 1:2, 1:4, 1:5, 1:10). The reaction mixtures in CD_2_Cl_2_ have been continuously analyzed by NMR spectroscopy over the course of 2 days (ESI, Figures S24–S27). Interestingly, the formation of compound **1** could not be observed spectroscopically at ratios of 1:5 and 1:10 and only a signal corresponding to BF_4_^−^ is visible in the ^19^F{^1^H} NMR spectrum. In the ^31^P{^1^H} NMR spectrum of the 1:5 approaches only one signal, which can be assigned to tetraphenyl diphosphine (Ph_2_PPPh_2_), is displayed. For the 1:10 approach, where more PPO is being present, additional signals for oxygen‐containing compounds, such as Ph_2_P(═O)P(═O)Ph_2_ and Ph_2_P(═O)–O–PPh_2_, can be identified. The formation of the oxygen‐containing phosphorus species cannot be monitored in the case of the 1:5 reaction, since these compounds most likely form paramagnetic complexes with Ni(II). In the 1:10 approach, the quantity of the ligands exceeds the available coordination‐sites at the nickel center and free Ph_2_P(═O)–O–PPh_2_ and Ph_2_P(═O)–P(═O)Ph_2_ compounds can be observed in the NMR spectra.

**Figure 4:**
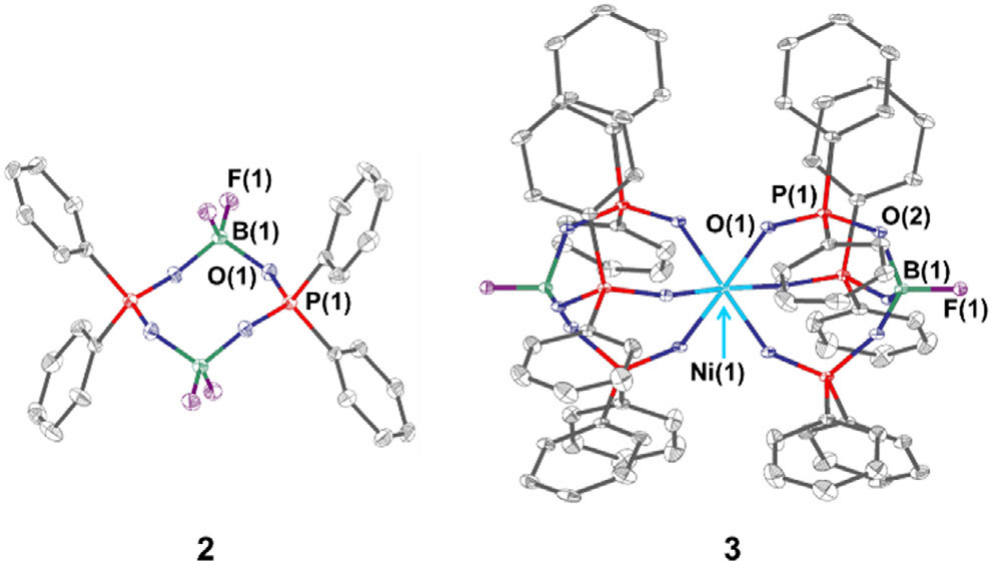
Molecular structures of the by‐products **2** and **3**. Hydrogen atoms are emitted for clarity and displacement ellipsoids are drawn at 30% probability. Selected bond lengths (Å) and angles (°): for **2**: C–P 1.7787(12)–1.7816(11) B–F 1.3586(14)–1.3800(15) B–O 1.4910(14)–1.4960(15) O–P 1.5314(8)–1.5360(8) F–B–O 108.25(9)–110.01(10) B–O–P 129.40(7)–129.81(7) C–P–C 110.32(5) O–P–O 115.16(5) for **3**: Ni–O 2.0501(13)–2.0502(13) B–F 1.369(4) C–P 1.793(2)–1.797(2) P–O 1.4867(13)–1.5566(14) O–B 1.4776(18) F–B–O 108.59(15)–108.59(15) B–O–P 132.46(15) O–P–O P–O–Ni 144.24(9).

At ratios with increasing amounts of Ni precursor (1:4, 1:2, and 1:1 ratios) the recorded ^31^P{^1^H} and ^19^F{^1^H} NMR spectra (Figure 4, for a 1:4 ratio) are similar and all show the successful formation of nickel complex **1**. In addition, the ^31^P{^1^H} NMR spectra indicate the presence of a PPO‐coordinated nickel species in which, according to the chemical shift of the Ph_2_P ═ O‐unit, the oxygen coordinates to the Ni(II) center. Furthermore, the formation of tetraphenyl diphosphine dioxide (Ph_2_P(═O)P(═O)Ph_2_, 26.4 ppm) and Ph_2_PPPh_2_ (–14.7 ppm) can be witnessed (ESI, Figures S24–S27). The respective amount of the two latter ones increases with higher quantities of PPO. At this point it must be underlined again, that due to the paramagnetic character of many Ni(II) species, some Ni(II)‐containing by‐products and intermediates might not be visible in the respective NMR spectra.

Based on the strong impact of the reaction stoichiometry on the product formation and the absence of any fluorinated phosphine species, if PPO is used in excess (at 1:5 and 1:10 ratios), we assume that two different reaction pathways compete, which either result in the formation of PFPh_2_ or of Ph_2_PPPh_2_. The second reaction channel is based on P─P bond cleavage of the PPO ligand and subsequent recombination reactions, forming Ph_2_PPPh_2,_ Ph_2_P(═O)–O–PPh_2_ and Ph_2_P(═O)P(═O)Ph_2_ in presence of the nickel precursor. Similar P─P bond activations were reported by Ogawa and co‐workers in presence of Rh or Pd compounds.^[^ ^43^, ^44^ ^]^ The second reaction pathway strongly dominates over the fluorination in an excess of the PPO ligand.

In approaches of 1:1, 1:2, and 1:4 ratios, after 1 day, the formation of another P‐ and F‐containing compound can be seen from the ^31^P{^1^H} and ^19^F{^1^H} NMR spectra (^31^P{^1^H} NMR: 32.7 ppm, q, ^3^ *J*_PF_ = 10.3 Hz and ^19^F{^1^H} NMR: 139.7 ppm, t, ^3^ *J*_PF_ = 10.1 Hz). This compound, namely Ph_2_PO_2_(BF_2_)_2_O_2_PPh_2_ (**2**), along with the nickel(II) complex [Ni(OPOPh_2_)_6_(BF)_2_] (**3**) and the targeted compound (**1**) could be co‐crystallized from a DCM/*n*‐pentane mixture and unambiguously identified by SCXRD. Hereby the quantity of **2** in the reaction solution is significantly increased with a longer reaction time (ESI, Figures S26–S27). Both compounds **2** and **3** indicate the stepwise defluorination of the BF_4_^−^ anion during the synthesis of **1**, as well as the formation of Ph_2_POO^−^ as residual phosphine oxide species.

The solid state structures of Ph_2_PO_2_(BF_2_)_2_O_2_PPh_2_ (**2**) and [Ni(OPOPh_2_)_6_(BF)_2_] (**3**) are given in Figure 4. In **2** a macrocycle of two BF_2_^+^ and two Ph_2_POO^−^ units was formed, in which two BF_2_^+^ units bridge the two ligands through a quasi‐tetrahedral, four‐coordinated boron center. Compound **3** represents a nickel(II) complex, in which the nickel center is octahedrally coordinated by six anionic Ph_2_POO^−^ ligands. On both sides of the complex, the ligand scaffold is capped by one BF unit. This compound was isolated before,^[^ ^45^ ^]^ however based on misinterpretation of the SCXRD data, the compound was falsely identified as [Ni(OP(H)Ph_2_)_6_(BF_4_)_2_] (further information can be found in the ESI). The isolation of compounds **2** and **3** is highly likely a result of their high stability, which is conferred by the four‐coordinated boron centers.^[^ ^46^ ^]^ Due to the insolubility of **1** in acetonitrile, the by‐products **2** and **3** could be easily separated from **1**. Efforts to reduce the by‐product formation via the adjustment of the speed of the addition, the modification of the reaction temperature or time have proven to be unsuccessful.

**Figure 5:**
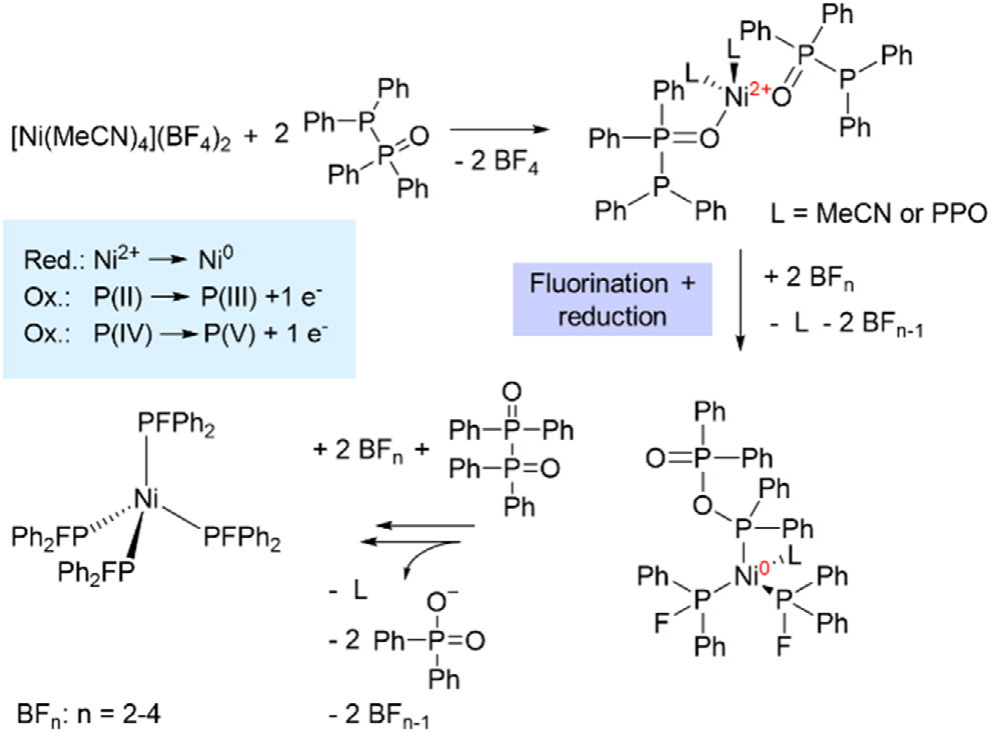
Simplified reaction mechanism for the formation of **1** via a fluorination and Ni(II) → Ni(0) reduction mechanism. F^−^ anions stem from the stepwise BF_4_^−^ decomposition.

Based on the identification of various by‐products, a distinctive reduction and fluorination mechanism is suggested in Figure 5. Firstly PPO coordinates to Ni(II) via the oxygen donor function, which weakens the P─P bond within the ligand scaffold. Through a simultaneous P─P bond cleavage and fluorination of the P atoms, electrons can then be transferred to the Ni(II) center, leading to the reduction to Ni(0), while the phosphorus atom is being partly oxidized (ESI, Figure S44). Subsequently, two Ph_2_P═O‐fragments, resulting from the P─P cleavage, recombine to form Ph_2_P(═O)–P(═O)Ph_2_. This compound undergoes a tautomeric equilibrium shift and coordinates via its P(III) donor atom to the Ni(0) center and can consequently be fluorinated again. From this reaction Ph_2_P(═O)_2_^−^ is released, which is observed in the isolated by‐products **2** and **3**. The last step is repeated to form the final product **1**. For this redox‐chemistry to occur, the uncommon oxidation states and the shift in between the different tautomers of PPO and Ph_2_P(═O)–P(═O)Ph_2_ are decisive (ESI, Figure S44).

It is crucial to emphasize that no evidence of a nickel fluoride species is detectable in the ^19^F{^1^H} NMR spectra of the reaction mixture (ESI, Figure S28). This eliminates the possibility of fluorophosphine ligand formation via a fluorine transfer mechanism. Such a pathway, previously reported by Macgregor, Grushin, and co‐workers for a fluorophosphine‐stabilized Rh complex, can therefore be excluded in the present system.^[^ ^32^, ^47^ ^]^

### Theoretical Investigations

To investigate the donor properties of the herein reported unexplored fluorophosphine ligand, PFPh_2_, the Tolman electronic parameter (TEP) was calculated using density functional theory (DFT, see ESI for further computational details), in line with the experimental method developed by Tolman.^[^ ^48^ ^]^

**Figure 6:**
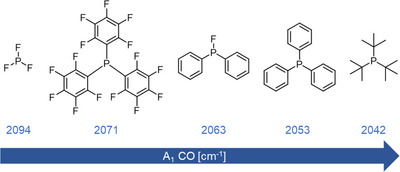
Computationally determined Tolman electronic parameters (B3LYP/def2‐TZVP).^[^ ^49^, ^50^, ^51^, ^52^ ^]^

From the calculated vibrations and the higher *ν*_CO_, which indicate a less electron rich metal center resulting from weaker phosphine donors, P(*^t^* Bu_3_) can be considered a solely *σ*‐donating phosphine, whereas PF_3_ is known to be a very strong π‐acceptor ligand (Figure 6). The fluorophosphine PFPh_2_ is found to exhibit an intermediate donor strength between the two extremes as mainly π‐accepting ligand, and therefore its application opens new avenues to tune the reactivity of metal complexes, especially with respect to catalytic applications.

**Figure 7:**
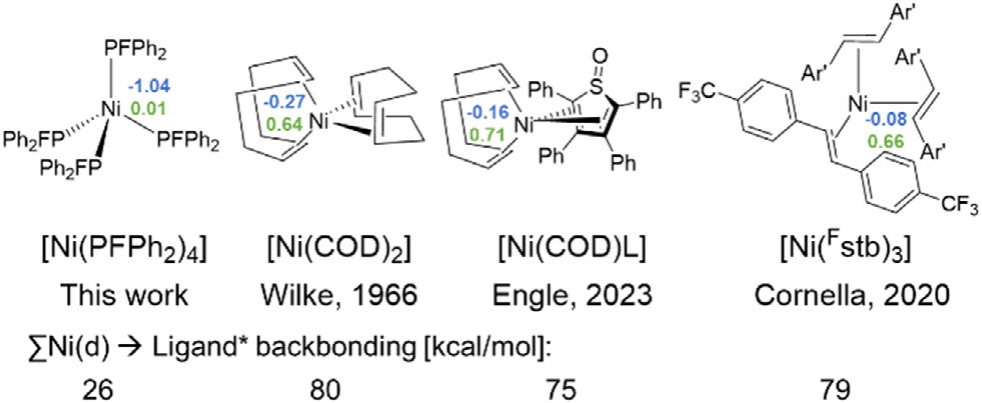
Computational investigation of **1** along with other state of the art Ni(0) complexes. Loewdin charges from Loewdin population analysis are marked in blue, whereas natural charges from natural population analysis are highlighted in green. In addition, the sum of the Ni(d) to ligand* backbonding, obtained from second order perturbation analysis, is shown (B3LYP/def2‐TZVP).^[^ ^49^, ^50^, ^51^, ^52^ ^]^

In addition to the investigation of the donor properties of the free PFPh_2_ ligand, DFT and population analyses were conducted for complex **1**. For a comparison with state of the art nickel(0) complexes, Wilke's [Ni(COD)_2_],^[^ ^53^ ^]^ [Ni(COD)L] (L═2,3,4,5‐tetraphenylthiophene‐1‐oxide) reported by Engle et al.,^[^ ^19^ ^]^ and [Ni(^F^stb)_3_] (^F^stb═trans‐1,2‐bis(4‐(trifluoromethyl)phenyl)ethene) described by Cornella et al.,^[^ ^20^ ^]^ were investigated (Figure 7).

Figure 7 shows the charges obtained from Loewdin (blue) and natural population analysis (green) along with the degree of backbonding from the nickel d‐orbitals into the anti‐bonding orbitals of the corresponding ligands. Whereas in all other complexes beside **1**, olefin ligands are present, which enable a high degree of backbonding (of about 75–80 kcal mol^−1^) and therefore cause an increase in charge at the Ni centers, the electronic character of the fluorophosphine renders the Ni center in **1** quite electron rich. This again indicates the potential of **1** to extend the family of bench‐stable Ni(0) pre‐catalysts with tailored electronic properties in the field of homogeneous catalysis.

### Stability Studies

Surprisingly, complex **1** in its solid‐state is stable for months under air and even in water. The decomposition point was determined to be around 160 °C, which is comparable to [Ni(COD)L] complexes, including quinones, cyclopentadienones, thiophene‐S‐oxides, and fulvenes.^[^ ^19^ ^]^ The complex can therefore be manipulated at ambient conditions without the need of Schlenk line techniques or glove boxes. The stability might result from the effective steric shielding of the nickel center by the ligands, as indicated by the space filling diagram of the complex (ESI, Figure S53).

Furthermore, the stability of **1** in solution has been investigated. In contrast to other [Ni(0)L_4_] complexes,^[^ ^54^, ^55^ ^]^ no ligand dissociation yielding a three‐coordinated complex, could be observed spectroscopically, even at elevated temperatures. Subsequently, the stability of **1** under UV light (365 nm) in solution was explored by NMR and UV/Vis measurements. These analytic techniques revealed the formation of dark nickel particles along with the disproportionation of the ligand^[^ ^29^ ^]^ forming PF_3_Ph_2_ and Ph_2_P–PF_2_Ph_2_ (ESI, Figures S29–S32 and Figure S52). This is in accordance with the calculated UV/Vis spectra of **1** and its decomposition products obtained from time‐dependent DFT (TD‐DFT, ESI, Figure S58).

**Figure 8:**
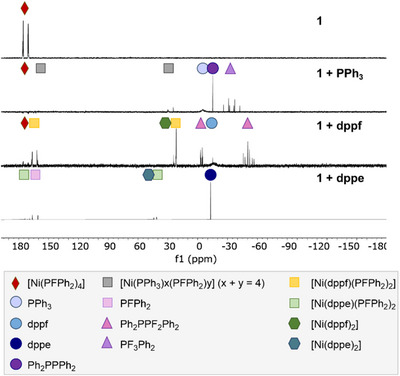
Investigation of ligand exchange with PPh_3_, dppf and dppe via ^31^P{^1^H} NMR spectroscopy in C_6_D_6_. For PPh_3_ and dppf, ligand exchange can be achieved through UV radiation (365 nm), while heat (100 °C) is sufficient in the case of dppe.

Furthermore, the reactivity of **1** toward ligand substitution has been studied by NMR spectroscopy (Figure 8), using 1,5‐cyclooctadiene (COD), triphenyl phosphine (PPh_3_), 1,2‐bis(diphenylphosphino)ethane (dppe), 1,1′‐bis(diphenyl‐phosphino)ferrocene (dppf) and 2,2′‐bis(diphenylphosphino)‐1,1′‐binaphthyl (BINAP). For the ligand exchange with PPh_3_ or dppf UV radiation (365 nm) is required. The NMR spectra show that the liberated fluorophosphine ligands disproportionate in solution. For the ligand substitution with dppe, a temperature of 100 °C is sufficient for the formation of [Ni(PFPh_2_)_2_(dppe)]. Interestingly, with dppe as well as with dppf only small quantities of [Ni(phosphine)_2_] species can be observed, which are often considered as catalysts sinks.^[^ ^13^ ^]^ Using COD and BINAP only very minor ligand substitution reactivity becomes obvious from the NMR spectra.

### Catalytic Activity

To the best of our knowledge, there is no example of the application of fluorophosphines as ligands in coupling reactions. Thus, our goal was to investigate the performance of the herein developed Ni(0) complex **1**, stabilized by unconventional fluorophosphine ligands, as (pre‐)catalyst in Suzuki–Miyaura coupling reactions (Table 1). Based on the stability studies presented before, PPh_3_, dppe and dppf were added to **1** along with the reactants and the solvent. The mixtures were then irradiated with UV light (365 nm, 15 min), to induce ligand exchange, and subsequently heated to the required temperature of 100 °C (detailed information can be found in the ESI).

**Table 1:** Suzuki–Miyaura coupling reactions of iodobenzene (1 eq.) and phenylboronic acid (1.5 eq.). Reaction conditions, if not noted otherwise: 2.5 mol% catalyst, 5 mol% additional ligand, 3 equiv. K_2_CO_3_, toluene as solvent and *n*‐decane as internal standard. Samples were heated to 100 °C for 14 h. Conversions of aryl halides were determined via GC‐MS. Product formation occurs with 100% selectivity. UV activation refers to 15 min of UV radiation (365 nm) of the reaction mixture before heating to 100 °C. r.t. = room temperature.

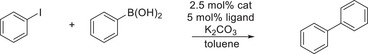				
Entry	Ni(0) source	Ligand	Conditions	Conversion (%)
1	**1**	–	r.t.	0
2	**1**	–	UV, r.t.	0
3	**1**	–	100 °C	60
4	**1**	–	UV, 100 °C	72
5	**1**	PPh_3_	UV, 100 °C	47
6	**1**	dppe	100 °C	22
7	**1**	dppe	UV, 100 °C	18
8	**1**	dppf	100 °C	80
9	**1**	dppf	UV	13
10	**1**	dppf	UV, 100 °C	100
11	**1** ^a)^	dppf	UV, 100 °C	81

^a)^ With addition of COD (2:1 COD:**1**).

As it can be seen from the coupling reactions of iodobenzene and phenylboronic acid, the addition of PPh_3_ and dppe leads to a drop of conversion (Table 1, entries 5 and 7). This might stem from a poisoning effect, due to the formation of a saturated Ni species. In contrast, the mixed‐ligand system of **1** and dppf shows significant potential as catalyst in Suzuki–Miyaura coupling reactions (Table 1, entries 8–11).

Since iodoarenes, are known to be the most reactive aryl halides (bond dissociation energies *D* [kcal mol^−1^]: Ph–I = 65; Ph–Br = 81; Ph–Cl = 96; Ph–F = 126),^[^ ^6^, ^56^ ^]^ the catalytic tests were extended toward less reactive analogues (Table 2). With bromobenzene, a similar behavior was observed as with iodobenzene, whereby the addition of dppf to **1** leads to the highest conversion (Table 2, entry 4), while the addition of PPh_3_ or dppe results in lower catalytic activity than with pure **1**. The substrate scope was extended toward the more challenging compound chloroanisole. Again, the combination of **1** with dppf has shown to be the lead catalyst system.

**Table 2:** Suzuki–Miyaura couplings of phenylboronic acid (1.5 eq.) and iodobenzene, bromobenzene or chloroanisole with and without ligand addition. Reaction conditions: 2.5 mol% catalyst, 5 mol% ligand, 3 equiv. K_2_CO_3_, toluene as solvent and *n*‐decane as internal standard. Samples were exposed to UV light (365 nm) for 15 mins and heated to 100 °C for 14 h. Conversions of aryl halides were determined via GC‐MS.

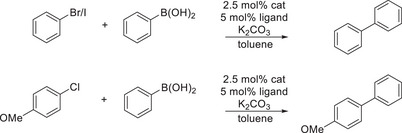					
Entry	Ni(0) source	Ligand	Conv. (%) I‐benzene	Conv. (%) Br‐benzene	Conv. (%) Cl‐anisole^a)^
1	**1**	–	72	82	21 (63)
2	**1**	PPh_3_	47	42	35 (92)
3	**1**	dppe	18	0	0
4	**1**	dppf	100	100	72 (95)
5	[Ni(COD)_2_]	–	15	0	0
6	[Ni(COD)_2_]	PPh_3_	31	27	24 (62)
7	[Ni(COD)_2_]	dppe	20	0	0
8	[Ni(COD)_2_]	dppf	82	52	56 (99)
9	–	–	0	0	0

^a)^ Selectivity toward 4‐methoxy biphenyl given in brackets.

In order to benchmark **1**, reference tests using [Ni(COD)_2_] instead of **1** as Ni(0) source have been conducted (Table 2, entries 5–8, ESI, Table S4). The comparison of the pure Ni(0) species (Table 2, entry 1 and 5) does already indicate an enhanced catalytic activity of **1** in comparison to [Ni(COD)_2_], which is further confirmed in the presence of additional ligands (Table 2, entries 6–8). These findings underline the potential of fluorophosphines as ligand class in the field of catalysis. One major disadvantage of [Ni(COD)_2_] as catalyst precursor is the blocking of active sites through free COD, resulting in deceleration of the catalysis.^[^ ^13^, ^57^, ^58^ ^]^ In contrast, the liberated fluorophosphine ligands of **1** are decomposed under UV light and therefore, do not tend to re‐coordinate to the Ni(0) center. This results in the generation of highly active Ni(0) species. The inhibition effect of COD was further demonstrated by the addition of COD to **1** and dppf (Table 1, entry 11).

To explore the potential of **1** to perform Suzuki–Miyaura coupling reactions chemoselectively, 4‐chloroanisole was selected as substrate. Hereby, the addition of an external phosphine was clearly recognized to cause an increase in selectivity (63% pure **1**; > 90% with addition of a phosphine, ESI, Table S5). If [Ni(COD)_2_] was used as Ni(0) precursor, a distinct difference of monodentate (62%) and bidentate (99%) phosphines was observed, while in reactions using **1**, no clear impact of the denticity was noticed (92% for PPh_3_ versus 95% for dppf, Table 2 and ESI, Table S5).

A screening of various substrates has been conducted to assess the functional group tolerance (ESI, Figure S56). In earlier studies, the catalytic conversion of α‐halo‐*N*‐heterocycles, such as 2‐chloropyridine or 2‐chloroquinoline, using a Ni/dppf‐based catalyst has been reported to be impeded, due to the formation of dimeric Ni‐species.^[^ ^4^ ^]^ In contrast, our **1**/dppf‐based lead system demonstrates good catalytic performance in the coupling of 2‐bromopyridine with phenylboronic acid, with no indication of a catalyst sink. Further, electron‐withdrawing groups are very well tolerated (see bromo‐acetophenone). However, the conversion of sterically hindered 2‐bromo xylene results in a decreased conversion. Excellent conversion and selectivity have also been achieved with *p*‐tolylboronic acid, while a chloro substituent in 4‐chloro phenylboronic acid results in poor catalytic activity.

**Scheme 1:**
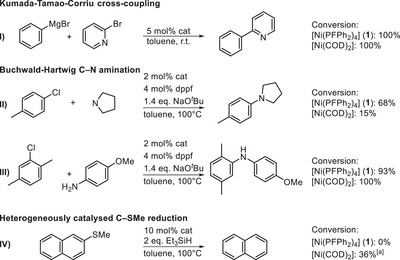
Performance of **1** in other catalytic reactions compared to [Ni(COD)_2_]. I) Aryl‐aryl Kumada‐Tamao‐Corriu cross‐coupling involving the coupling of a heterocycle, II) Buchwald–Hartwig C─N bond formation of an alkylamine, III) Buchwald–Hartwig C─N bond formation of an arylamine, IV) C─SMe reduction catalyzed by forming Ni nanoparticles. [a]: if the reaction was conducted in the presence of dppf (20 mol%) no conversion was observed.

To further underscore the potential of **1** as alternative Ni(0) source, its performance was evaluated in Kumada‐Tamao‐Corriu cross‐coupling reactions between 2‐bromopyridine and PhMgBr. Notably, hereby no catalyst deactivation induced by the heterocycle was observed (Scheme 1(I)). Additionally, in Buchwald–Hartwig C─N bond formations with both alkyl‐ (Scheme 1(II)) and arylamines (Scheme 1(III)), **1** demonstrated comparable or even superior conversions compared to [Ni(COD)_2_]. The enhanced stability of **1** relative to [Ni(COD)_2_] was further evident in the catalytic activation of C─S bonds (Scheme 1(IV)). Unlike [Ni(COD)_2_], which forms catalytically active Ni(0) nanoparticles in the absence of a ligand,^[^ ^20^ ^]^ no such behavior was observed for [Ni(PFPh_2_)_4_] (**1**). Consequently, **1** did not facilitate catalytic conversion in this case. These findings demonstrate that using **1** instead of [Ni(COD)_2_] effectively prevents undesired decomposition reactions into heterogeneous nanoparticles, due to its enhanced stability.

### Catalyst Poisoning Studies

To confirm the homogeneous nature of our nickel fluorophosphine‐based pre‐catalyst, even after UV radiation, we attempted poisoning experiments using dct (dibenzo [a,e]cyclooctatetraene, further information can be found in the ESI, Table S6), which is known to poison homogeneous catalytic species. However, contrary to other studies in which 2 equiv. of dct per metal atom were shown to inhibit the catalysis completely,^[^ ^59^, ^60^, ^61^ ^]^ the addition of 4 eq. of dct still resulted in a conversion of 35%. Consequently, it can be assumed that dct might not be the most efficient poison for this system and that other experiments are needed to verify the findings.^[^ ^62^, ^63^ ^]^ This is in accordance with earlier studies, which found high conversions with homogeneous catalysts in presence of dct for Suzuki–Miyaura coupling reactions.^[^ ^64^ ^]^

Therefore, another poisoning test was carried out using CS_2_, which is especially useful for stoichiometric poisoning of homogeneous and sub‐stoichiometric poisoning of heterogeneous catalysts.^[^ ^63^ ^]^ Hereby, sub‐stochiometric amounts of CS_2_ did not cause complete catalytic inhibition as it would be expected for heterogeneous catalysts. Further, the addition of 2 equiv. of CS_2_ to the reaction mixture of **1** and dppf, has shown a conversion of 15%, indicating a homogeneous nature of the catalytic system (ESI, Table S6).

For further clarification, a filtration test according to Maitlis et al.^[^ ^65^ ^]^ was performed (ESI, Table S7). Hereby, equal conversions can be obtained for the filtrate and the control experiment, which was conducted as usual. This result confirms the homogeneous nature of the catalytically active Ni(0) species.

## Conclusion

In conclusion, we report the synthesis of a fluorophosphine‐stabilized nickel(0) complex [Ni(PFPh_2_)_4_] (**1**) via a unique in situ reduction and fluorination mechanism. The remarkable air‐ and moisture stability of the complex in its solid state paves the way toward the application of **1** as bench‐stable pre‐catalyst in coupling reactions. Especially, the combination of the fluorophosphine‐based complex **1** together with dppf as ligand after a tailored UV/heat activation procedure results in remarkable catalytic properties in various reactions, which can be exploited to activate a wide substrate scope. In contrast to [Ni(COD)_2_], for which dissociated COD ligands can inhibit the catalysis, the liberated fluorophosphine ligand of [Ni(PFPh_2_)_4_] can be decomposed under UV light. This way blocking of the active sites can completely be prevented. This study offers rare insights into the class of fluorophosphines as ligands for transition metals applied in the field of catalysis. Further research will be focused on a targeted ligand modification to uncover unknown ligand effects, to tailor the ligand substitution on the nickel center and to explore the promising catalytical potential of such transition metal complexes further.

## Supporting Information

Experimental details, analytic as well as catalytic data is given in the Supporting Information of this article. CCDC 2379067 (**1**), 2379068 (**2**), and 2379069 (**3**) contain the supplementary crystallographic data for this paper. These data can be obtained free of charge via www.ccdc.cam.ac.uk/data_request/cif, or by emailing data_request@ccdc.cam.ac.uk, or by contacting The Cambridge Crystallographic Data Center, 12 Union Road, Cambridge CB2 1EZ, UK; fax: +44 1223 336033. The authors have cited additional references within the Supporting Information.^[^ ^66^, ^67^, ^68^, ^69^, ^70^, ^71^, ^72^, ^73^, ^74^, ^75^, ^76^, ^77^, ^78^, ^79^, ^80^, ^81^, ^82^, ^83^, ^84^, ^85^, ^86^, ^87^, ^88^, ^89^, ^90^, ^91^, ^92^, ^93^, ^94^, ^95^, ^96^, ^97^, ^98^, ^99^, ^100^ ^]^ The data that support the findings of this study are openly available in Zenodo at https://doi.org/10.5281/zenodo.13691978, reference number.^[^ ^101^ ^]^

## Conflict of Interests

The authors declare no conflict of interest.

## Supporting information



Supporting Information S1

Supporting Information S2

Supporting Information S3

Supporting Information S4

## Data Availability

The data that support the findings of this study are openly available in [Zenodo] at [10.5281/zenodo.13691978], reference number [13691978].

## References

[1] V. M. Chernyshev , V. P. Ananikov , ACS Catal. 2022, 12, 1180–1200.

[2] V. P. Ananikov , ACS Catal. 2015, 5, 1964–1971.

[3] S. Z. Tasker , E. A. Standley , T. F. Jamison , Nature 2014, 509, 299–309. 24828188 10.1038/nature13274 PMC4344729

[4] A. K. Cooper , M. E. Greaves , W. Donohoe , P. M. Burton , T. O. Ronson , A. R. Kennedy , D. J. Nelson , Chem. Sci. 2021, 12, 14074–14082. 34760191 10.1039/d1sc04582b PMC8565371

[5] A. K. Cooper , D. K. Leonard , S. Bajo , P. M. Burton , D. J. Nelson , Chem. Sci. 2020, 11, 1905–1911. 34123283 10.1039/c9sc05444h PMC8148322

[6] F.‐S. Han , Chem. Soc. Rev. 2013, 42, 5270. 23460083 10.1039/c3cs35521g

[7] J. B. Diccianni , T. Diao , Trends Chem 2019, 1, 830–844.

[8] Z. Li , L. Liu , Chinese J. Catal. 2015, 36, 3–14.

[9] S.‐S. Wang , G.‐Y. Yang , Catal. Sci. Technol. 2016, 6, 2862–2876.

[10] X. Hu , Chem. Sci. 2011, 2, 1867.

[11] C. Zhu , H. Yue , J. Jia , M. Rueping , Angew. Chem. Int. Ed. 2021, 60, 17810–17831. 10.1002/anie.202013852 33252192

[12] M. S. Oderinde , N. H. Jones , A. Juneau , M. Frenette , B. Aquila , S. Tentarelli , D. W. Robbins , J. W. Johannes , Angew. Chem. Int. Ed. 2016, 55, 13219–13223. 10.1002/anie.201604429 27436532

[13] A. L. Clevenger , R. M. Stolley , J. Aderibigbe , J. Louie , Chem. Rev. 2020, 120, 6124–6196. 32491839 10.1021/acs.chemrev.9b00682

[14] E. A. Standley , S. J. Smith , P. Müller , T. F. Jamison , Organometallics 2014, 33, 2012–2018. 24803717 10.1021/om500156q PMC4006606

[15] E. Richmond , J. Moran , Synth. 2018, 50, 499–513.

[16] V. T. Tran , Z.‐Q. Li , O. Apolinar , J. Derosa , M. V. Joannou , S. R. Wisniewski , M. D. Eastgate , K. M. Engle , Angew. Chem. Int. Ed. 2020, 59, 7409–7413. 10.1002/anie.202000124 32065839

[17] E. A. Standley , T. F. Jamison , J. Am. Chem. Soc. 2013, 135, 1585–1592. 23316879 10.1021/ja3116718 PMC3564234

[18] J. B. Sweeney , A. K. Ball , P. A. Lawrence , M. C. Sinclair , L. J. Smith , Angew. Chem. Int. Ed. 2018, 57, 10202–10206. 10.1002/anie.201805611 29939450

[19] V. T. Tran , N. Kim , C. Z. Rubel , X. Wu , T. Kang , T. C. Jankins , Z.‐Q. Li , M. V. Joannou , S. Ayers , M. Gembicky , J. Bailey , E. J. Sturgell , B. B. Sanchez , J. S. Chen , S. Lin , M. D. Eastgate , S. R. Wisniewski , K. M. Engle , Angew. Chem. Int. Ed. 2023, 62, e202211794. 10.1002/anie.202211794 PMC9987410 36524997

[20] L. Nattmann , R. Saeb , N. Nöthling , J. Cornella , Nat. Catal. 2020, 3, 6–13.

[21] L. Nattmann , J. Cornella , Organometallics 2020, 39, 3295–3300.

[22] C. Compain , B. Donnadieu , F. Mathey , Organometallics 2006, 25, 540–543.

[23] R. J. O'Reilly , A. Karton , Front. Chem. 2023, 11, 1283418. 37854977 10.3389/fchem.2023.1283418 PMC10579588

[24] A. B. Burg , Inorg. Nucl. Chem. Letters 1977, 13, 199–203.

[25] M. Fild , R. Schmutzler , J. Chem. Soc. A. 1970, 2359–2364.

[26] P. M. Miura‐Akagi , T. W. Chapp , W. Y. Yoshida , G. P. A. Yap , A. L. Rheingold , R. P. Hughes , M. F. Cain , Organometallics 2023, 42, 672–688.

[27] T. Mizuta , T. Yamasaki , H. Nakazawa , K. Miyoshi , Organometallics 1996, 15, 1093–1100.

[28] W. S. Sheldrick , O. Stelzer , J. Chem. Soc. Dalton Trans. 1973, 926–929.

[29] L. Heuer , P. G. Jones , R. Schmutzler , J. Fluor. Chem. 1990, 46, 243–254.

[30] N. Fey , M. Garland , J. P. Hopewell , C. L. McMullin , S. Mastroianni , A. G. Orpen , P. G. Pringle , Angew. Chem. Int. Ed. 2012, 51, 118–122. 10.1002/anie.201105954 22076744

[31] A. M. Miles‐Hobbs , P. G. Pringle , J. D. Woollins , D. Good , Molecules 2024, 29, 2368. 38792229 10.3390/molecules29102368 PMC11123747

[32] V. V. Grushin , W. J. Marshall , J. Am. Chem. Soc. 2004, 126, 3068–3069. 15012134 10.1021/ja049844z

[33] F. C. Bradley , E. H. Wong , Inorg. Chim. Acta 1986, 120, L21–L22.

[34] A. W. Kyri , G. Schnakenburg , R. Streubel , Organometallics 2017, 36, 3605–3612.

[35] R. H. Morris , J. F. Sawyer , C. T. Schweitzer , A. Sella , Organometallics 1989, 8, 2099–2106.

[36] F. D. Calvo , V. Mirabello , M. Caporali , W. Oberhauser , K. Raltchev , K. Karaghiosoff , M. Peruzzini , Dalton Trans. 2016, 45, 2284–2293. 26732238 10.1039/c5dt04624f

[37] S. Hanf , T. Grell , J. E. Waters , R. García‐Rodríguez , E. Hey‐Hawkins , D. S. Wright , Chem. Commun. 2020, 56, 7893–7896. 10.1039/d0cc02142c 32524101

[38] J. Reedijk , Comments Inorganic Chem 1982, 1, 379–389.

[39] R. F. Jordan , W. E. Dasher , S. F. Echols , J. Am. Chem. Soc. 1986, 108, 1718–1719.

[40] M. R. Mason , J. G. Verkade , Organometallics 1990, 9, 864–865.

[41] P. A. McLaughlin , J. G. Verkade , Organometallics 1998, 17, 5937–5940.

[42] M. R. Mason , J. G. Verkade , Organometallics 1992, 11, 2212–2220.

[43] S.‐i. Kawaguchi , M. Kotani , T. Ohe , S. Nagata , A. Nomoto , M. Sonoda , A. Ogawa , Phosphorus Sulfur Silicon Relat. Elem. 2010, 185, 1090–1097.

[44] Y. Yamamoto , K. Fujiwara , A. Ogawa , Organometallics 2023, 42, 2590–2597.

[45] A. F. Gushwa , Y. Belabassi , J.‐L. Montchamp , A. F. Richards , J. Chem. Crystallogr. 2009, 39, 337–347.

[46] D. Delgado , R. Abonia , Arab. J. Chem. 2022, 15, 103528.

[47] S. A. Macgregor , D. C. Roe , W. J. Marshall , K. M. Bloch , V. I. Bakhmutov , V. V. Grushin , J. Am. Chem. Soc. 2005, 127, 15304–15321. 16248673 10.1021/ja054506z

[48] C. A. Tolman , Chem. Rev. 1977, 77, 313–348.

[49] A. D. Becke , J. Phys. Chem. 1993, 98, 5648–5652.

[50] P. J. Stephens , F. J. Devlin , C. F. Chabalowski , M. J. Frisch , J. Chem. Phys. 1994, 98, 11623–11627.

[51] F. Weigend , Phys. Chem. Chem. Phys. 2006, 8, 1057. 16633586 10.1039/b515623h

[52] F. Weigend , R. Ahlrichs , Phys. Chem. Chem. Phys. 2005, 7, 3297. 16240044 10.1039/b508541a

[53] B. Bogdanović , M. Kröner , G. Wilke , Liebigs Ann. Chem. 1966, 699, 1–23. 10.1002/jlac.19666990102 5986842

[54] C. A. Tolman , W. C. Seidel , L. W. Gosser , J. Am. Chem. Soc. 1974, 96, 53–60.

[55] J. F. Nixon , R. Schmutzler , Spectrochim. Acta A 1964, 20, 1835–1842.

[56] V. V. Grushin , H. Alper , Chem. Rev. 1994, 94, 1047–1062.

[57] A. J. Nett , W. Zhao , P. M. Zimmerman , J. Montgomery , J. Am. Chem. Soc. 2015, 137, 7636–7639. 26057139 10.1021/jacs.5b04548

[58] D. D. Dawson , V. F. Oswald , A. S. Borovik , E. R. Jarvo , Chem.‐Eur. J. 2020, 26, 3044–3048. 31953874 10.1002/chem.202000215 PMC7872209

[59] A. M. A. Boshaala , S. J. Simpson , J. Autschbach , S. Zheng , Inorg. Chem. 2008, 47, 9279–9292. 18808107 10.1021/ic800611h

[60] P. Büschelberger , D. Gärtner , E. Reyes‐Rodriguez , F. Kreyenschmidt , K. Koszinowski , A. J. v. Wangelin , R. Wolf , Chem.‐Eur. J. 2017, 23, 3139–3151. 28026060 10.1002/chem.201605222 PMC5861671

[61] D. Gärtner , A. Welther , B. R. Rad , R. Wolf , A. J. V. Wangelin , Angew. Chem. Int. Ed. 2014, 53, 3722–3726. 10.1002/anie.201308967 24616276

[62] J. A. Widegren , R. G. Finke , J. Mol. Catal. A Chem. 2003, 198, 317–341.

[63] D. Gärtner , S. Sandl , A. J. v. Wangelin , Catal. Sci. Technol. 2020, 10, 3502–3514.

[64] S. Baweja , T. Gabler , P. Lönnecke , E. Hey‐Hawkins , Dalton Trans. 2023, 52, 6494–6500. 37096400 10.1039/d3dt00507k

[65] J. Hamlin , K. Hirai , A. Millan , P. M. Maitlis , J. Mol. Catal. A Chem. 1980, 7, 543–544.

[66] A. Sen , T.‐W. Lai , R. R. Thomas , J. Organomet. Chem. 1988, 358, 567–588.

[67] Stoe&Cie , in *Darmstadt, Germany, Vol. X‐RED, Program for data reduction and absorption correction*, Program for data reduction and absorption correction 1.28b ed., Stoe & Cie GmbH Darmstadt, Germany 2005, pp. X‐RED, Program for data reduction and absorption correction.

[68] G. M. Sheldrick , Acta. Cryst. B 2015, 71, 3–8. 10.1107/S2053273314026370 PMC4283466 25537383

[69] G. M. Sheldrick , Acta. Cryst. A 2015, 71, 3–8. 10.1107/S2053273314026370 PMC4283466 25537383

[70] L. J. Farrugia , J. Appl. Crystallogr. 2012, 45, 849–854.

[71] K. Brandenburg , M. Berndt , in Diamond, 4.6.8 ed., Crystal Impact , Bonn, Germany 1999, pp. Diamond–Crystal and Molecular Structure Visualization.

[72] Y. Morino , K. Kuchitsu , T. Moritani , Inorg. Chem. 1969, 8, 867–871.

[73] H. Oberhammer , R. Schmutzler , O. Stelzer , Inorg. Chem. 1978, 17, 1254–1258.

[74] D. Gudat , A. Haghverdi , H. Hupfer , M. Nieger , Chem.‐Eur. J. 2000, 6, 3414–3425. 11039535 10.1002/1521-3765(20000915)6:18<3414::aid-chem3414>3.0.co;2-p

[75] M. W. Bezpalko , B. M. Foxman , C. M. Thomas , Inorg. Chem. 2015, 54, 8717–8726. 26302438 10.1021/acs.inorgchem.5b01363

[76] F. Neese , WIREs Comput. Mol. Sci. 2017, 8, e1327.

[77] F. Neese , WIREs Comput. Mol. Sci. 2012, 2, 73–78.

[78] F. Neese , F. Wennmohs , A. Hansen , U. Becker , Chem. Phys. 2009, 356, 98–109.

[79] S. Grimme , J. Antony , S. Ehrlich , H. Krieg , J. Phys. Chem. 2010, 132, 154104. 10.1063/1.3382344 20423165

[80] S. Grimme , S. Ehrlich , L. Goerigk , J. Comp. Chem. 2011, 32, 1456–1465. 21370243 10.1002/jcc.21759

[81] J. Tao , J. P. Perdew , V. N. Staroverov , G. E. Scuseria , Phys. Rev. Lett. 2003, 91, 146401–146404. 14611541 10.1103/PhysRevLett.91.146401

[82] V. N. Staroverov , G. E. Scuseria , J. Tao , J. P. Perdew , J. Phys. Chem. 2004, 121, 11507. 10.1063/1.1665298 15267588

[83] C. M. Breneman , K. B. Wiberg , J. Comp. Chem. 1990, 11, 361–373.

[84] E. D. Glendening , J. K. Badenhoop , A. E. Reed , J. E. Carpenter , J. A. Bohmann , C. M. Morales , P. Karafiloglou , C. R. Landis , F. Weinhold , Theoretical Chemistry Institute, University of Wisconsin, Madison 2018, p. NBO 7.0.

[85] A. E. Reed , L. A. Curtiss , F. Weinhold , Chem. Rev. 1988, 88, 899–926.

[86] A. E. Reed , R. B. Weinstock , F. Weinhold , J. Phys. Chem. 1985, 83, 735–746.

[87] C. Adamo , D. Jacquemin , Chem. Soc. Rev. 2013, 42, 845–856. 23117144 10.1039/c2cs35394f

[88] A. B. Burg , G. B. Street , Inorg. Chem. 1966, 5, 1532–1537.

[89] A. Manzoor , P. Wienefeld , M. C. Baird , P. H. M. Budzelaar , Organometallics 2017, 36, 3508–3519.

[90] in DAISY, part of TopSpin 4.1.1, Bruker BioSpin GmbH, Rheinstetten 2020.

[91] L. Riesel , J. Haenel , Z. Anorg. Allg. Chem. 1991, 603, 145–150.

[92] L. Riesel , J. Haenel , G. Ohms , J. Fluor. Chem. 1988, 38, 335–340.

[93] L. Riesel , D. Sturm , A. Nagel , S. Taudien , A. Beuster , A. Karwatzki , Z. Anorg. Allg. Chem. 1986, 542, 157–166.

[94] L. P. Miller , J. A. Vogel , S. Harel , J. M. Krussman , P. R. Melvin , Org. Lett. 2023, 25, 1834–1838. 36897224 10.1021/acs.orglett.3c00274 PMC10043933

[95] S. Hanf , A. L. Colebatch , P. Stehr , R. García‐Rodríguez , E. Hey‐Hawkins , D. S. Wright , Dalton Trans. 2020, 49, 5312–5322. 32242884 10.1039/d0dt00609b

[96] T. M. Maier , S. Sandl , P. Melzl , J. Zweck , A. J. V. Wangelin , R. Wolf , Chem.‐Eur. J. 2020, 26, 6113–6117. 32034810 10.1002/chem.201905537 PMC7318650

[97] D. R. Anton , R. H. Crabtree , Organometallics 1983, 2, 855–859.

[98] F. L. Hirshfeld , Acta. Cryst. A 1976, A32, 239–244.

[99] G. Mavel , Ann. Rep. NMR Spectr. 1973, 5B, 1.

[100] J. P. Wolfe , S. L. Buchwald , Eds., J. Am. Chem. Soc. 1997, 119, 6054–6058.

[101] F. Flecken , A. Neyyathala , T. Grell , S. Hanf , Zenodo 2025, 10.5281/zenodo.13691978. PMC12144898 40202383

