# Supplementary material for: A Bench‐Stable Fluorophosphine Nickel(0) Complex and Its Catalytic Application
Source: Angew Chem Int Ed Engl. 2025 Apr 18;64(24):e202506271. doi: 10.1002/anie.202506271 (PMC12144898; doi:10.1002/anie.202506271)
Supplement: Supplementary file 1 — Supporting Information S1 [file ANIE-64-e202506271-s001.docx]

A Bench-Stable Fluorophosphine Nickel(0) Complex and Its Catalytic Application

Franziska Flecken,^[a]^ Arjun Neyyathala,^[a]^ Toni Grell^[b]^ and Schirin Hanf*^[a]^

^[a]^ Karlsruhe Institute of Technology, Institute for Inorganic Chemistry, Engesserstr. 15, 76131 Karlsruhe, Germany

E-mail: schirin.hanf@kit.edu

^[b]^ Dipartimento di Chimica, Institution Università degli Studi di Milano, Via Camillo Golgi 19, 20131 Milano, Italy

Data sets in open file formats have been deposited on Zenodo (DOI: 10.5281/zenodo.13691978)

[1. Experimental section 1](#_Toc193119897)

[2. Stability studies 5](#_Toc193119898)

[3. NMR spectra 6](#_Toc193119899)

[4. Further characterisation of compound 1 30](#_Toc193119901)

[5. X-ray Crystallographic details 34](#_Toc193119902)

[6. Comparison with reported P–F containing compounds 38](#_Toc193119903)

[7. Catalytic tests 40](#_Toc193119904)

[8. Poisoning experiments 43](#_Toc193119905)

[9. Computational details 45](#_Toc193119906)

[10. Literature 51](#_Toc193119907)

# Experimental section

All experiments were carried out under Argon using Schlenk-line techniques and an Argon-filled glove box (MBraun). Dichloromethane (DCM) and acetonitrile (MeCN) were freshly distilled over calcium hydride. Toluene, *n*-heptane and tetrahydrofuran (THF) were obtained from a MBraun solvent purification system (SPS-800) and degassed before use. THF was further distilled over potassium/benzophenone. Deuterated C_6_D_6_, CD_3_CN and d_8_-toluene were dried over CaH_2_ and CDCl_3_ and CD_2_Cl_2_ were dried over P_2_O_5_. Deuterated solvents were further prepared by three freeze-pump-thaw cycles. All solvents were stored over molecular sieves (3 Å for MeCN, all other solvents over 4 Å). The PPO/POP ligand was synthesized according to literature procedures.scp All other chemicals were used without further purification.

NMR spectra were recorded on a Bruker Avance III or Avance Neo 400 MHz at 298 K, if not noted otherwise, whereby the chemical shifts are referred to residual solvent signals of the deuterated solvents and are given in parts per million (ppm). By means of chemical shifts, coupling patterns and 2D NMR experiments (^1^H–^1^H COSY, ^1^H–^13^C HMQC) peaks were assigned. Following abbreviations were used for the multiplicity: s = singlet, d = doublet, dd = doublet of doublets, t = triplet, sep = septet, m = multiplet, br = broad. NMR spectra were evaluated using MestreNova.

UV/Vis spectra were recorded using an Ocean FX UV-Vis spectrometer from Ocean Optics. Therefore, solutions with a concentration of c = 5·10^-5^ mol/L were prepared. The data was processed with the program Origin. For irradiation of the samples with UV light (365 nm) a UV lamp (UVGL-15) of Analytik Jena was utilised.

Elemental analyses of the samples were conducted with a vario EL cube or a vario MICRO cube (Elementar Analysensysteme GmbH).

IR spectra were obtained using a Bruker Tensor 37 FTIR spectrometer equipped with a room temperature DLaTGS detector and a diamond ATR (attenuated total reflection) unit. The spectrometer contains a nitrogen filled chamber. IR spectra were recorded in the region of 4000-400 cm^-1^. Signal intensities were divided into vs = very strong, s = strong, m = medium, w = weak, vw = very weak.

For electron paramagnetic resonance (EPR) spectroscopic measurements a Bruker EMXplus spectrometer was used, whereby the field was calibrated against 2,2-diphenyl-1-picrylhydrazine (DDPH) with a *g* value of 2.0036.

Electrospray Ionization Mass Spectrometry (ESI-MS) was performed on a Fisher Scientific LTQ Orbitrap XLQ Exactive. All signals are referred to the mass-to-charge ratio (m/z).

Gas chromatography–mass spectrometry (GC-MS) was conducted using an Agilent 8860 GC and a 5977B MSD.

**Synthesis of [Ni(MeCN)_4_](BF_4_)_2_**

[Ni(MeCN)_4_](BF_4_)_2_ was synthesized according to modified literature procedures.^[1]^ A suitable possibility to relief the overpressure has to be ensured during the whole course of the reaction. A suspension of 2 g of NOBF_4_ in MeCN was added to 0.5 g of Ni powder in MeCN. The reaction can be initiated by heat and after stirring of the reaction mixture over night, subsequent filtration and addition of diethyl ether, the reaction mixture is cooled down to 4°C for successful crystallization of purple-blue crystals.

**Synthesis of [Ni(PFPh_2_)_4_] (1)**

Compound **1** was synthesized by the addition of the PPO ligand (109.0 mg, 0.282 mmol, 2 eq.) to [Ni(MeCN)_4_](BF_4_)_2_ (56.0 mg, 0.141 mmol) in DCM (5 mL). The reaction mixture turned blue-greenish immediately and shortly afterwards to a brown colour. After 2 hours the reaction mixture was filtered and layered with MeCN. Overnight yellow needle-like crystals suitable for single crystals XRD were formed (30.6 mg, 0.035 mmol, 25% crystalline yield).

m.p./b.p.: At 161°C **1** starts to decompose in the solid state

^1^H NMR (400 MHz, C_6_D_6_, ppm): *δ* = 7.53 (br, 16 H, Ar-H-*meta*), 6.92 (br, 16 H, Ar-H-*ortho*), 6.91 (br, 8H; Ar-H-*para*)

^31^P{^1^H} NMR (162 MHz, C_6_D_6_, ppm): *δ* = 173.1 (AA’A’’A’’’XX’X’’X’’’, ^2^*J*_PP_ = –23.26 Hz, ^1^*J*_PF_ = –850.25 Hz, ^3^*J*_PF_ = +45.50 Hz)

^13^C{^1^H} NMR (101 MHz, C_6_D_6_, ppm): *δ* = 130.3 (br, Ar-C-*meta*), 129.0 (s, Ar-C-*para*), 127.7 (s, Ar-C-*ortho*, signal is superimposed by solvent)

^13^C{^1^H} NMR (101 MHz, CD_2_Cl_2_, ppm): *δ* = 130.0 (br, Ar-C-*meta*), 129.5 (s, Ar-C-*para*), 128.0 (s, Ar-C-*ortho*)

^19^F{^1^H} NMR (377 MHz, C_6_D_6_, ppm): *δ* = –134.5 (AA’A’’A’’’XX’X’’X’’’, ^1^*J*_PF_ = –850.25 Hz, ^3^*J*_PF_ = +45.50 Hz, ^4^*J*_FF_ = –1.4 Hz)

^11^B NMR (96 MHz, C_6_D_6_, ppm) *δ* = no signal

ATR-IR (cm^−1^): $\tilde{v}$ = 3542(w), 3051(w), 3015(vw), 2948(vw), 2917(w), 2846(vw), 1548(vw), 1571(w), 1478(w), 1431(m), 1397(vw), 1373(vw), 1310(w), 1276(vw), 1234(w), 1182(vw), 1154(w), 1127(vw), 1098(s), 1068(w), 1023(w), 997(w), 981(w), 927(vw), 912(vw), 858(vw), 754(w), 743(w), 730(vs, P-F stretching), 692(vs), 615(w), 523(s), 489(m), 459(m), 435(m)

UV/Vis (dcm, nm (mol^−1^dm^3^cm^−1^)): *λ*_max_ (*ε*) = 310 (6600), 235 (10200)

HR-MS (ESI): *m*/*z* calculated for [C_48_H_40_F_4_NiP_4_+H]^+^: 875.14483 [*M*]; found: 875.13739

Elemental analysis calculated (%) for C_48_H_40_F_4_NiP_4_: C 65.86, H 4.61 found: C 66.11, H 4.228.

**Synthesis of [Ph_2_PO_2_(BF_2_)_2_O_2_PPh_2_] (2)**

Compound **2** can be co-crystallized with compound **1**, if the layering is performed with a DCM/*n*-pentane mixture. **2** is formed together with compound **3**. It should be underlined, that the formation of **2** is also observed if the reaction is conducted in an Argon-filled glovebox. Thereby, it can be verified that **2** is a byproduct during the synthesis of **1** and does not stem from any air contamination.

^1^H and ^13^C NMR spectra are disturbed by paramagnetic character of 3.

^31^P{^1^H} NMR (162 MHz, CDCl_3_, ppm): *δ* = 33.6 (m, ^3^*J*_PF_ = 10.9 Hz)

^19^F{^1^H} NMR (377 MHz, CDCl_3_, ppm): *δ* = –139.9 (m, ^3^*J*_PF_ = 10.9 Hz)

^11^B NMR (96 MHz, CDCl_3_, ppm): *δ* = –0.5

It should be noted that in ^31^P and ^19^F NMR spectra, the formation of Ph_2_P(=O)F can be observed, which is due to deoxyfluorination of Ph2POO^–^.

**Synthesis of [Ni(OPOPh_2_)_6_(BF)_2_] (3)**

Compound **3** can be obtained as a by-product in the conversion of PPO with [Ni(MeCN)_4_](BF_4_)_2_ yielding **1**, through layering a DCM solution with *n*-pentane. Compounds **2** and **3** are simultaneously isolated from this solvent mixture. In analogy to **2**, compound **3** is also formed if the reaction takes place in an Argon-filled glovebox. Thus, it can be verified that **3** is a by-product during the synthesis of **1** and does not result from any air contamination. However, a selective formation of **3** can be achieved through controlled addition of air to the reaction mixture of the PPO ligand and [Ni(MeCN)_4_](BF_4_)_2_ in DCM. Pale yellow crystals can be obtained through filtering the reaction solution after 5 hours of reaction time and layering with *n*-heptane.

No ^1^H, ^31^P and ^13^C signals can be detected due to paramagnetic character.

^19^F{^1^H} NMR (377 MHz, CDCl_3_, ppm): *δ* = –147.2 (d, ^1^*J*_BF_ = 11.0 Hz)

^11^B NMR (96 MHz, CDCl_3_, ppm): *δ* = –0.8

**Isolation of Ph_2_PPPh_2_ (4)**

Ph_2_PPPh_2_ was isolated as a by-product of the synthesis of **1**. After separation of the crystals of **1**, the solvent of the supernatant was reduced *in vacuo* up to a sufficient precipitation of a colorless solid (**4**). The brownish supernatant was filtered off and the colourless powder of **4** was carefully washed with MeCN and subsequently dried *in vacuo*. NMR analysis of the reaction confirmed that the formed compound is Ph_2_PPPh_2_.

^1^H NMR (400 MHz, CD_3_CN, ppm): *δ*=7.48–7.41 (m, 8 H, Ar-H-*meta*), 7.35–7.21 (m, 12 H, Ar-H-*ortho/para*)

^31^P{^1^H} NMR (162 MHz, CD_3_CN, ppm): *δ* = –18.1 (s)

^13^C NMR (101 MHz, CD_3_CN, ppm): *δ* = 136.7 (br, Ar-C-*meta*), 135.2 (m, Ar-C-*meta), 130.1* (br, Ar-C-*ortho*/*para*), 129.5 (br, Ar-C-*ortho*/*para*)

^19^F{^1^H} NMR (377 MHz, CD_3_CN, ppm): *δ*= no signal

^11^B NMR (96 MHz, CD_3_CN, ppm): *δ* = no signal

Elemental analysis calculated (%) for C_24_H_20_P_2_: C 77.83, H 5.44 found: C 78.39, H 4.918

**Catalytic tests**

All catalytic tests have been conducted under Argon atmosphere either in Young-NMR tubes or in Schlenk-flasks equipped with a stirring bar.

*Suzuki-Miyaura coupling reactions*

The substrates aryl halide (0.1 mmol) and phenyl boronic acid (1.5 eq., 0.15 mmol), catalyst (2.5 mol%), base (3 eq., 0.3 mmol), ligands (5 mol%) and *n*-decane (10 μL) as the internal standard have been dissolved in toluene (0.6 mL). Various activation modes for the catalyst have been applied and the reaction mixture was heated to the required temperature. After completion of the reaction time, the reaction mixture was allowed to cool down to room temperature and filtered off. The products (100 μL) were analysed by GC-MS using an Agilent 8860 GC and a 5977B MSD in 1 mL MeCN or EtOH. MeCN can be beneficial since the catalyst is not soluble in this solvent.

*Kumada-Tamao-Corriu cross-coupling reactions*

Bromopyridine (0.05 mmol) and PhMgBr (phenyl magnesium bromide 1 M in THF, 1 eq., 0.05 mmol) were added to a solution of the catalyst (5 mol%) in toluene (0.5 mL) containing the internal standard *n*-decane (10 μL). To ensure that **1** is dissolved, the sample containing **1** was heated to 80°C for 10 minutes prior to the addition of the reagents. After 20 h of reaction time at room temperature the samples were filtered and analysed using GC-MS (100 μL sample in 1 mL EtOH).

*Buchwald-Hartwig C–N amination*

Buchwald-Hartwig aminations were conducted based on the procedure described by Buchwald.^[2]^ 1 eq. of aryl chloride was converted with the amine (1.2 eq.) in presence of NaO*^t^*Bu (1.4 eq.) in toluene (0.5 mL) containing *n*-decane as the internal standard. The C–N amination of alkyl amines was conducted using 4-chlorotoluene (0.25 mmol) and pyrrolidine (0.30 mmol) whereas for the coupling of the aryl amine *p*-anisidine (0.12 mmol) 2-chloro-*p*-xylene (0.10 mmol) was applied. 2 mol% [Ni(COD)_2_] or [Ni(PFPh_2_)_4_] (**1**) and 1,1'-bis(diphenylphosphino)ferrocene (dppf) as ligand (4 mol%) were used as catalysts. The sample containing **1** was irradiated with UV light (365 nm) for 15 minutes prior to heating. The reaction mixture was heated to 100°C for 16 h and afterwards filtered and analysed using GC-MS (100 μL sample in 1 mL EtOH).

*C–SMe reduction catalysed by forming Ni nanoparticles*

For a C–S bond activation, 2-(methylthio)naphtalene (0.13 mmol) was reacted with Et_3_SiH (2 eq., 0.25 mmol) in toluene (0.5 mL) containing *n*-decane (20 μL) as the internal standard. The reaction was performed using [Ni(COD)_2_] or **1** as catalyst in absence and presence of an additional ligand (20 mol%). After 14 h at 100°C, the reaction mixture was filtered and analysed using GC-MS (100 μL sample in 1 mL EtOH).

# Stability studies

Ligand exchange studies were conducted using 4 equivalents of monodentate or 2 equivalents of bidentate ligands in C_6_D_6_ and were analysed at room temperature, at 100°C and after UV radiation (365 nm) using NMR spectroscopy. Following ligands were selected for the substitution studies: 1,5-cyclooctadiene (COD), triphenyl phosphine (PPh_3_), 1,2-bis(diphenylphosphino)ethane (dppe), 1,1'-bis(diphenylphosphino)ferrocene (dppf) and 2,2‘-bis(diphenylphosphino)-1,1’-binaphthyl (BINAP). With COD, almost no formation of a COD-coordinated Ni(0) species, e.g. [Ni(COD)_2_], was observed after heating and UV radiation. Only the ^19^F{^1^H} NMR spectrum of the solution after UV radiation shows minor additional signals in the area of **1**, which might be due to partial ligand detachment (Figure S36). Similar observations were made when BINAP was added to a solution of **1**. After UV radiation, PF_3_Ph_2_ and Ph_2_P–PF_2_Ph_2_ as decomposition products of the PFPh_2_ ligand stemming from **1,** in addition to free BINAP and trace amounts of mixed-ligand complexes could be observed (Figure S32-S33).

While almost no ligand substitution was observed for COD and BINAP, the application of PPh_3_, dppf or dppe changes the picture. When PPh_3_ was used as additional ligand and the reaction mixture was irradiated with light, a broadened signal of free PPh_3_ (–5 ppm) and coordinated PPh_3_ (25 ppm) in the ^31^P{^1^H} NMR spectrum point towards coordination and dynamic ligand exchange.^[3]^ In addition to these signals, new peaks can be observed in the region of **1** in the ^31^P{^1^H} NMR (around 170 ppm) and ^19^F{^1^H} NMR (ca. –135 ppm) spectra, which are the result of mixed-ligand complexes of the form [Ni(PFPh_2_)_x_(PPh_3_)_y_]. Further, the formation of PF_3_Ph_2_ (^31^P{^1^H} NMR: –34.8 ppm, 2t, ^1^*J*_PF, eq_ = 973.1 Hz, ^1^*J*_PF, ax_ = 832.4 Hz; ^19^F{^1^H} NMR: –34.1 ppm, dd, ^1^*J*_PF, ax_ = 832.4 Hz, ^2^*J*_FF_ = 39.5 Hz, –79.8 ppm, dt, ^1^*J*_PF, eq_ = 973.1 Hz, ^2^*J*_FF_ = 39.5 Hz) and Ph_2_P–PPh_2_ (^31^P{^1^H} NMR: –14.9 ppm, s) is observed. With dppf, UV light initiates the formation of [Ni(PFPh_2_)_2_(dppf)] (^31^P{^1^H} NMR: 164.0 ppm, m; 21.7 ppm, m; ^19^F{^1^H} NMR: –130.4 ppm, m) and traces of [Ni(dppf)_2_] (^31^P{^1^H} NMR: 24.2 ppm, s). Besides, the formation of Ph_2_P–PF_2_Ph_2_ is witnessed (^31^P{^1^H} NMR: –4.1 ppm, dt, ^1^*J*_PaPb_ = 269.1 Hz, ^2^*J*_PaF_ = 43.3 Hz; –51.4 ppm, 2t, ^1^*J*_PaPb_ = 269.1 Hz, ^1^*J*_PbF_ = 724.3 Hz; ^19^F{^1^H} NMR: –35.1 ppm, dd, ^2^*J*_PaF_ = 43.3 Hz, ^1^*J*_PbF_ = 724.3 Hz), which results from the disproportionation of the liberated PFPh_2_ ligand. In accordance with the mechanism reported by Riesel and Haenel,^[4]^ the disproportionation of two PFPh_2_ molecules leads to Ph_2_P–PF_2_Ph_2_ as observed with dppf, while the reaction with a third PFPh_2_ molecule triggers the formation of Ph_2_P–PPh_2_ and Ph_2_PF_3_ as in the case of PPh_3_.

In the case of dppe, a temperature of 100°C is sufficient to achieve ligand substitution yielding [Ni(PFPh_2_)_2_(dppe)] (^31^P{^1^H} NMR: 171.2 ppm, m; 41.4 ppm, m; ^19^F{^1^H} NMR: –128.6 ppm, m) and traces of [Ni(dppe)_2_] (^31^P{^1^H} NMR: 44.1 ppm, s). Surprisingly, also the free and intact PFPh_2_ ligand can be identified in the ^31^P{^1^H} NMR (163.5 ppm, d, ^1^*J*_PF_ = 884.3 Hz) and the ^19^F{^1^H} NMR (–195.4 ppm, d, ^1^*J*_PF_ = 884.3 Hz) spectra, due to the softer activation procedure using heat instead of UV light radiation. Both signals are significantly upfield shifted in comparison to the coordinated PFPh_2_ ligand bound in **1**.

# NMR spectra

Automated line-shape analysis of NMR spectra was done using Daisy under Bruker’s TopSpin^[5]^ version 3.6.5 (BRUKER, BioSpin GmbH, Rheinstetten) setting the absolute sign for ^1^*J*_PF_ negative as this was determined for similar compounds. For further information please see ^[6]^ and the references therein.

The point group symmetry of complex **1** in the solid state is *S*_4_, however assuming a free rotation of the phenyl groups the symmetry observed in the time-averaged NMR-experiment would be *T_d_*. Both point group symmetrisations in combination with the spin system AA’A’’A’’’XX’X’’X’’’ were considered for refining the spectrum. The model with tetrahedral symmetry *T_d_* gave decisively better *R* values which strongly indicates that the phenyl groups indeed rotate about the P−C axis (Figure S 10 and Figure S 11). The ^31^P{^1^H} NMR spectrum was refined for extracting the coupling constants 173.1 ppm (^2^*J*_PP_ = 23.26 Hz, ^1^*J*_PF_ = 850.25 Hz, ^3^*J*_PF_ = 45.50 Hz) in the ^31^P{^1^H} NMR spectrum as well as a multiplet at –134.6 ppm (^1^*J*_PF_ = 850.25 Hz, ^3^*J*_PF_ = 45.50 Hz, ^4^*J*_FF_ = 1.4 Hz) in the ^19^F{^1^H} spectrum (Figure S 2 and Figure S 4).

| Table S 1. ^31^P{^1^H} NMR parameters of 1. | | | |
| --- | --- | --- | --- |
| *Δ* [ppm] | *J*_PP_ [Hz] |  | *H* [Hz] |
| *δ* = –173.1 | ^2^*J*_PP_ = −23.26(5) |  | *H* = 4.0(1) |
|  | ^1^*J*_PF_ = −850.25(5) |  |  |
|  | ^3^*J*_PF_ = +45.50(3) |  |  |
|  | ^4^*J*_FF_ = −1.4(3) |  |  |
| *δ*, chemical shift; *J*, coupling constant; *H*, spectral half width. | | | |

Figure S 1. ^1^H NMR (298 K, C_6_D_6_, 400 MHz) spectrum of 1.


Figure S 2. ^31^P{^1^H} NMR (298 K, C_6_D_6_, 162 MHz) spectrum of **1** at room temperature.

Figure S 3. ^31^P{^1^H} NMR (324 K, C_6_D_6_, 162 MHz) spectrum of **1** at 50°C.


Figure S 4. ^19^F{^1^H} NMR (298 K, C_6_D_6_, 377 MHz) spectrum of **1**.

Figure S 5. ^13^C{^1^H} NMR (298 K, C_6_D_6_, 101 MHz) spectrum of **1**.


Figure S 6. ^13^C{^1^H} NMR (298 K, CD_2_Cl_2_, 101 MHz) spectrum of **1**.

Figure S 7. ^11^B NMR (298 K, C_6_D_6_, 96 MHz) spectrum of **1**.

Figure S 8. COSY NMR (298 K, C_6_D_6_, 400 MHz) spectrum of **1**.

Figure S 9. ^1^H-^13^C HMQC NMR (298 K, C_6_D_6_, 400 MHz, 101 MHz MHz) spectrum of **1**.


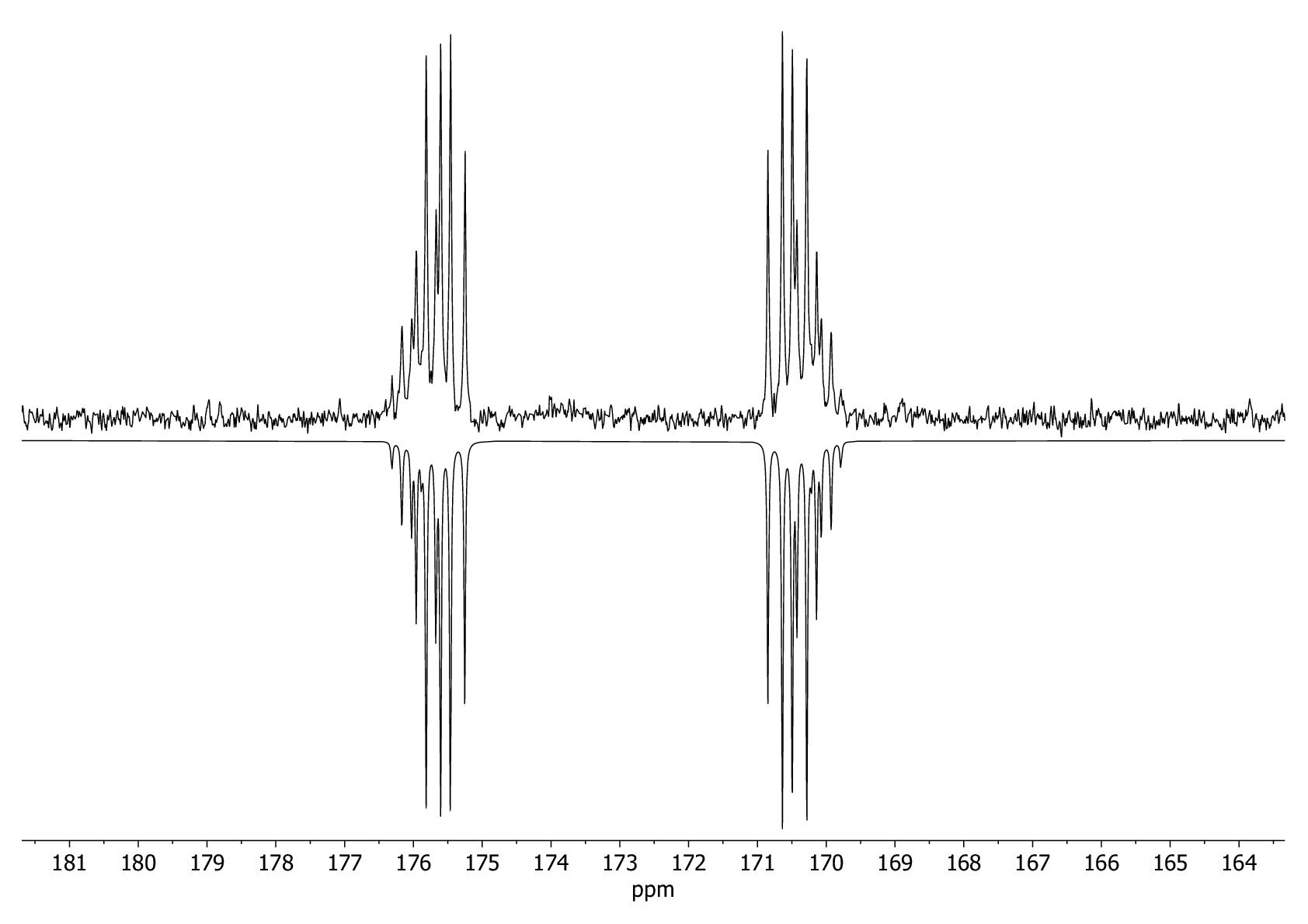


Figure S 10. Experimental (top) in C_6_D_6_ and simulated (bottom) ^31^P{^1^H} spectrum showing the AA’A’’A’’’XX’X’’X’’’ spin system of **1**.


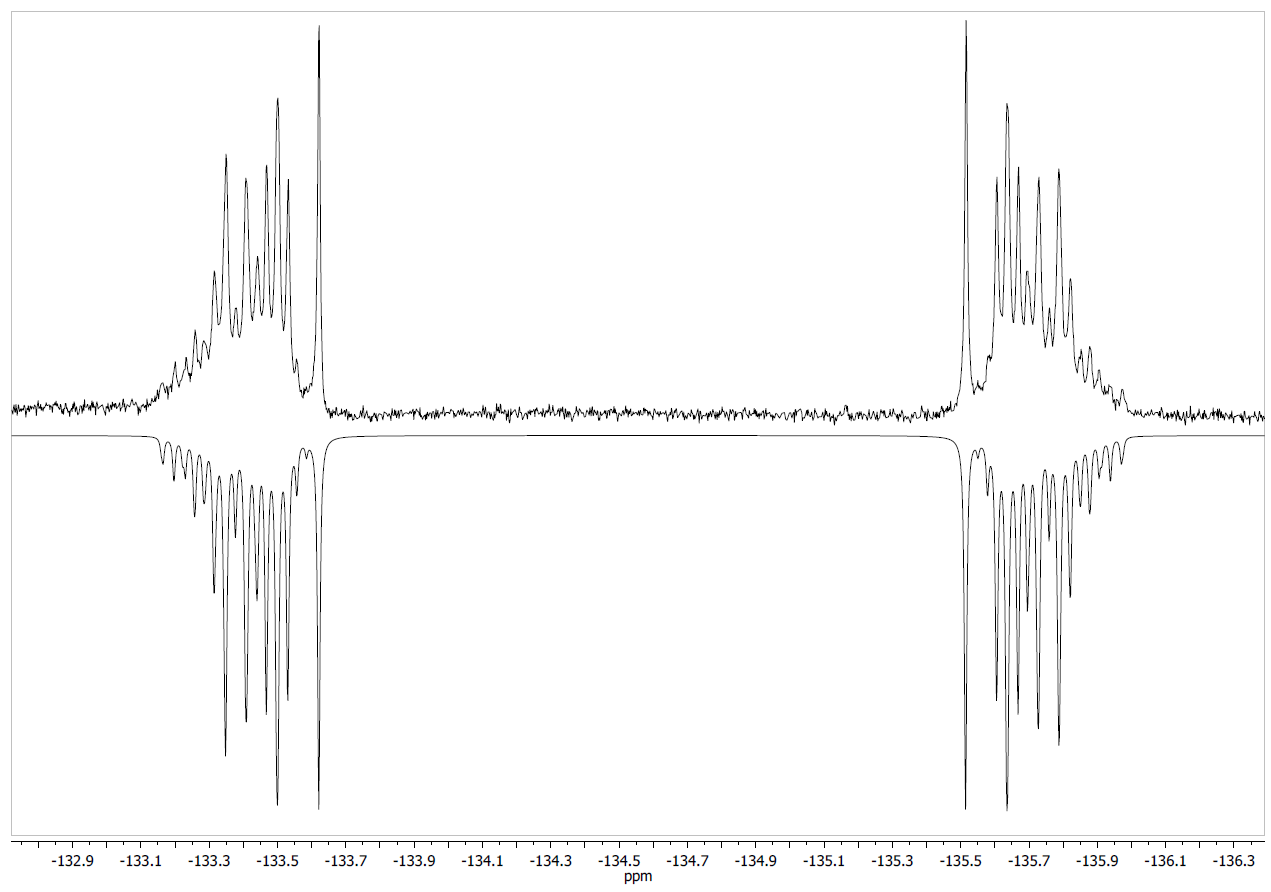


Figure S 11. Experimental (top) in C_6_D_6_ and simulated (bottom) ^19^F{^1^H} spectrum showing the AA’A’’A’’’XX’X’’X’’’ spin system of **1**.

Figure S 12. ^31^P{^1^H} NMR (298 K, CDCl_3_, 162 MHz) spectrum of a mixture of **2** (33.6 ppm, m, ^3^*J*_PF_ = 10.9 Hz) and **3** (paramagnetic). The formation of Ph_2_P(=O)F is observed (40 ppm, d, ^1^*J*_PF_ = 1020 Hz), which is highly likely due to deoxyfluorination of Ph_2_POO^–^ as reported by Miller *et al*., in which the deoxyfluorination of Ph_2_POOH in the presence of a base and a fluorination agent yields Ph_2_P(=O)F.^[7]^. The signal at 18.3 ppm is assigned to free Ph_2_POO^–^.

Figure S 13. ^19^F{^1^H} NMR (298 K, CDCl_3_, 377 MHz) spectrum of a mixture of **2** (–139.9 ppm, m, ^3^*J*_PF_ = 10.9 Hz) and 3 (–147.2 ppm, s). The formation of Ph_2_P(=O)F is observed (–75.2 ppm, d, ^1^*J*_PF_ = 1020 Hz) which is due to deoxyfluorination of Ph_2_POO^–^.

Figure S 14. ^11^B NMR (298 K, CDCl_3_, 96 MHz) spectrum of a mixture of **2** (–0.5 ppm) and **3** (–0.8 ppm).

Figure S 15. ^1^H NMR (298 K, CD_3_CN, 400 MHz) spectrum of Ph_2_PPPh_2_.


Figure S 16. ^31^P{^1^H} NMR (298 K, CD_3_CN, 162 MHz) spectrum of Ph_2_PPPh_2_.

Figure S 17. ^13^C{^1^H} NMR (298 K, CD_3_CN, 101 MHz) spectrum of Ph_2_PPPh_2_.


Figure S 18. ^19^F{^1^H} NMR (298 K, CD_3_CN, 377 MHz) spectrum of Ph_2_PPPh_2_.

Figure S 19. ^11^B NMR (298 K, CD_3_CN, 96 MHz) spectrum of Ph_2_PPPh_2_.


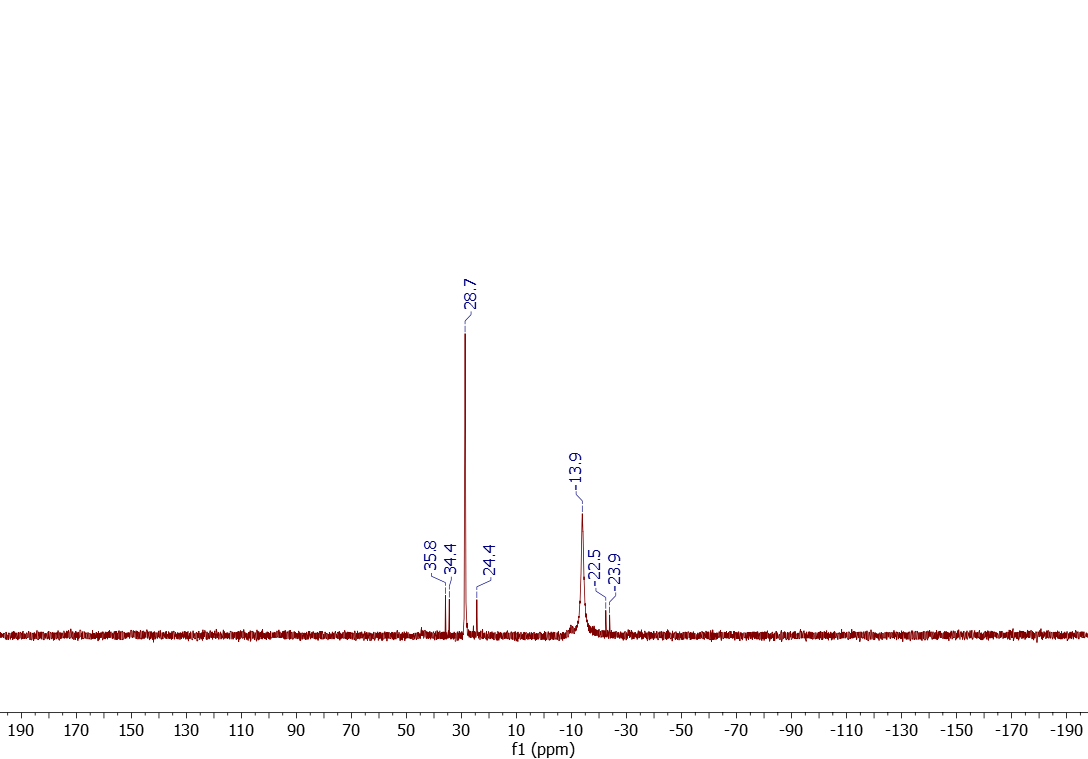


Figure S 20. ^31^P{^1^H} NMR (298 K, CD_2_Cl_2_, 162 MHz) spectrum of [Cu(MeCN)_4_](BF_4_)_2_ + PPO in CD_2_Cl_2_. No formation of a fluorinated species can be observed. However, formation of Ph_2_PPPh_2_ and Ph_2_P(=O)P(=O)Ph_2_ is detected.

Figure S 21. ^19^F{^1^H} NMR (298 K, C_6_D_6_, 377 MHz) spectrum of [Cu(MeCN)_4_](BF_4_)_2_ + PPO in CD_2_Cl_2_.


Figure S 22. ^31^P{^1^H} NMR (298K, CD_2_Cl_2_, 162 MHz) spectrum of NiOTf_2_ + PPO + NEt_4_BF_4_ in CD_2_Cl_2_. No conversion can be observed. Signals can be assigned to PPO (35.8 ppm, d, ^1^*J*_PP_ = 227.7 Hz, P=O; –22.5 ppm, d, ^1^*J*_PP_ = 227.7 Hz, P). Trace amounts of Ph_2_PPPh_2_ are observed (–15.2 ppm).

Figure S 23. ^19^F{^1^H} NMR (298 K, C_6_D_6_, 377 MHz) spectrum of NiOTf**_2_** + PPO + NEt_4_BF_4_ in CD_2_Cl_2_. No conversion can be observed. Signals can be assigned to the OTf^–^ anion at –78.5 ppm and the BF_4_^–^ anion at –152.3 ppm.


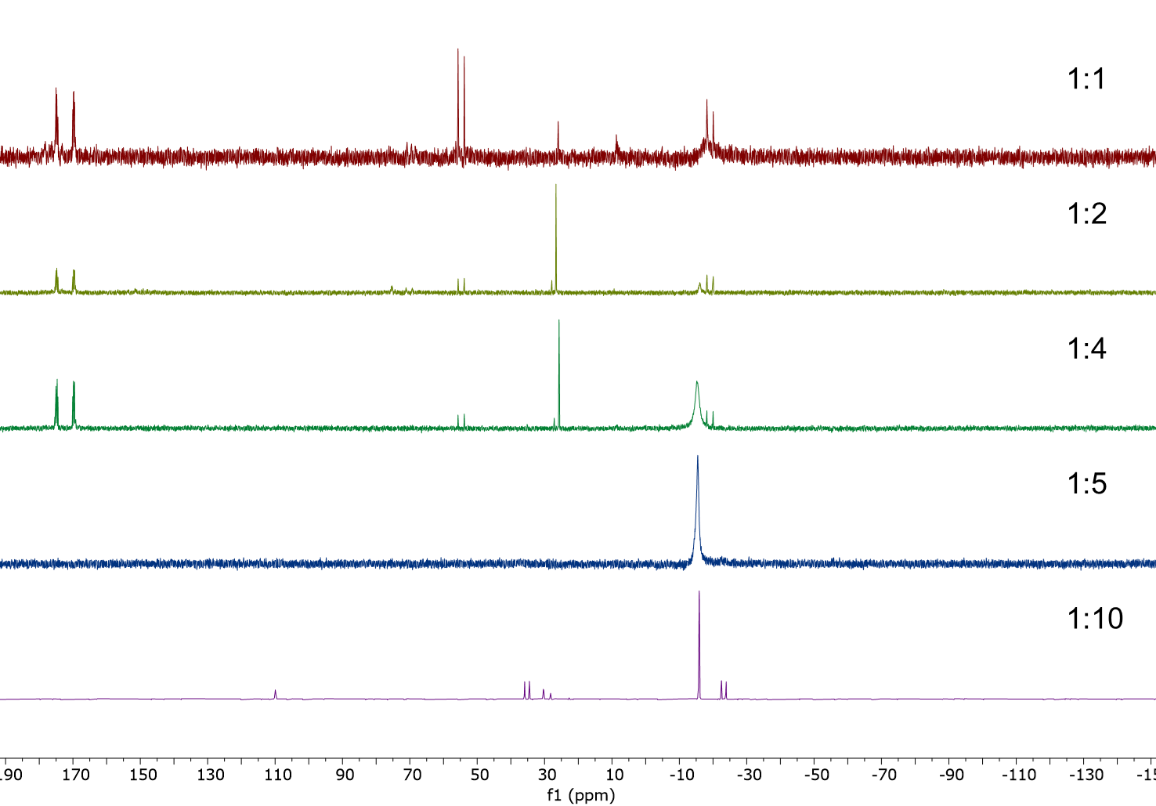


Figure S 24. ^31^P{^1^H} NMR (298 K, CD_2_Cl_2_, 162 MHz) spectra of various ratios of [Ni(MeCN)_4_](BF_4_)_2_ and PPO in CD_2_Cl_2_. The following compounds can be detected: **1** (173.1 ppm, m), PPO-ligated nickel species (55.5 ppm, d, ^1^*J*_PP_ = 301.7 Hz; 18.4 ppm, d, ^1^*J*_PP_ = 301.7 Hz), free PPO (35.8 ppm, d, ^1^*J*_PP_ = 227.7 Hz, P=O; –22.5 ppm, d, ^1^*J*_PP_ = 227.7 Hz, P), Ph_2_P(=O)P(=O)Ph_2_ (26.4 ppm, s), Ph_2_P(=O)–O–PPh_2_ (111.0 ppm, d, ^2^*J*_PP_ = 24.1 Hz; 31.5 ppm, d, ^2^*J*_PP_ = 24.1 Hz), Ph_2_PPPh_2_ (–14.7 ppm, s).


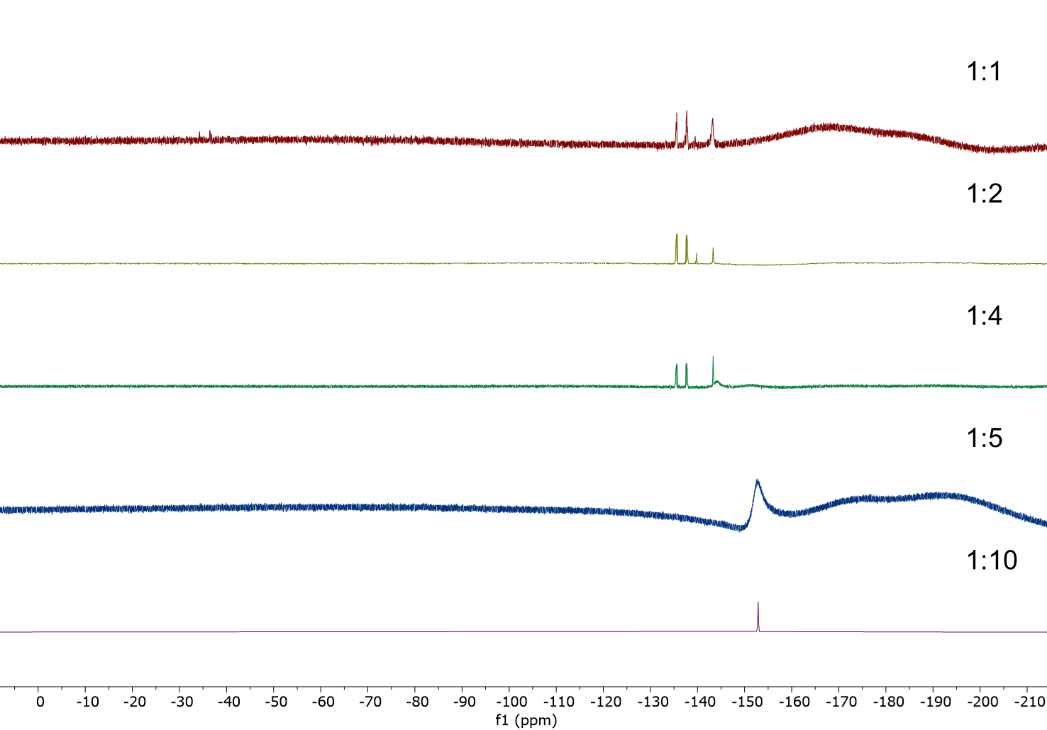


Figure S 25. ^19^F{^1^H} NMR (298K, C6D6, 377 MHz) spectra of various ratios of [Ni(MeCN)_4_](BF_4_)_2_ and PPO in CD_2_Cl_2_. The following compounds can be assigned: 1 (–134.6 ppm, m), BF_4_^–^ (–152.3 ppm, s). Signals at around –140 ppm cannot clearly be assigned to a specific compound but are assumed to be other B_x_F_y_ species.


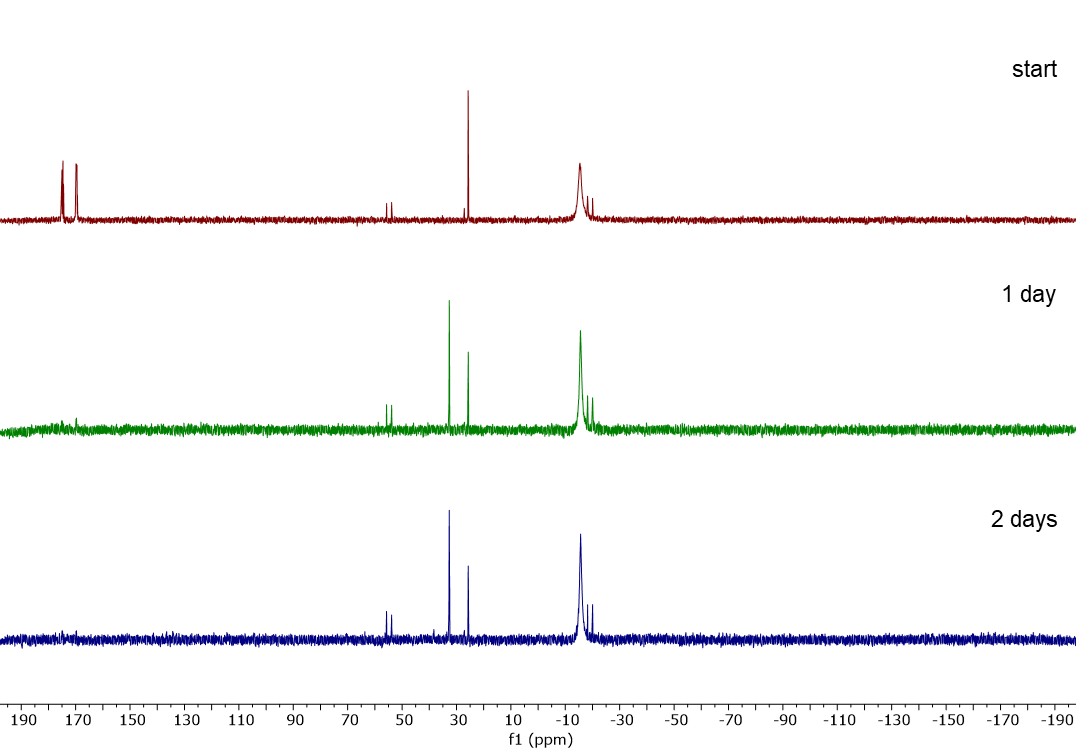


Figure S 26. ^31^P{^1^H} NMR (298 K, CD_2_Cl_2_, 162 MHz) spectra of the synthesis of **1** using a ratio of **1** [Ni(MeCN)_4_](BF_4_)_2_ and 4 PPO in CD_2_Cl_2_ over time. The following compounds can be detected: **1** (173.1 ppm, m), PPO-ligated Nickel species (55.5 ppm, d, ^1^*J*_PP_ = 301.7 Hz;, –18.4 ppm, d, ^1^*J*_PP_ = 301.7 Hz), after one day: by-product **2** (32.7 ppm, q, ^3^*J*_PF_ = 10.3 Hz; ^2^*J*_PB_ = 9.8 Hz), Ph_2_P(=O)P(=O)Ph_2_ (26.4 ppm, s), Ph_2_P–PPh_2_ (–14.7 ppm, s). The amount of **1** in the NMR spectra decreases with time which is due to solubility reasons since crystallization of 1 can be observed. By heating the sample, **1** can be brought back into solution.


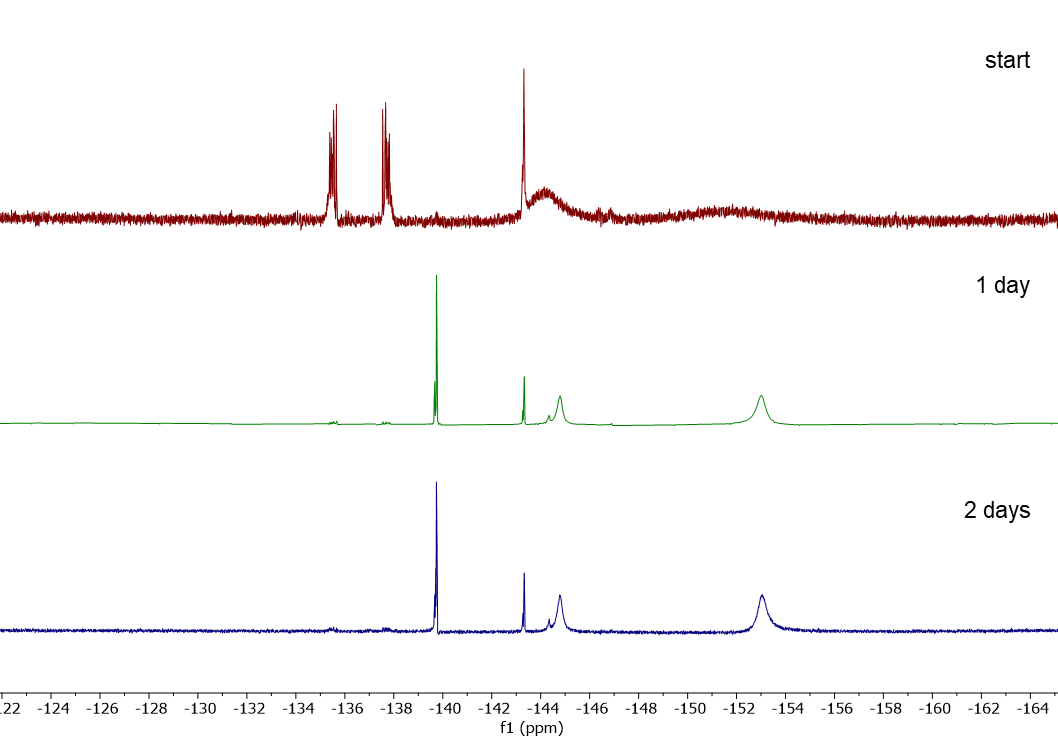


Figure S 27. ^19^F{^1^H} NMR (298 K, CD_2_Cl_2_, 377 MHz) spectrum of synthesis of **1** using a ratio of 1 [Ni(MeCN)_4_](BF_4_)_2_ and 4 PPO in CD_2_Cl_2_ over time. The following compounds can be detected: **1** (–134.6 ppm, m), after one day: by-product **2** (139.7 ppm, t, ^3^*J*_PF_ = 10.1 Hz), signals at around –140 ppm and –153 ppm cannot clearly be assigned to a specific compound but are assumed to be other B_x_F_y_ species. The amount of 1 in the NMR spectra decreases with time which is due to solubility reasons since crystallization of 1 can be observed. By heating the sample, 1 can be brought back into solution.

Figure S 28. ^19^F{^1^H} NMR (298 K, CD_2_Cl_2_, 377 MHz) spectrum of the synthesis of **1** using a ratio of 1 [Ni(MeCN)_4_](BF_4_)_2_ and 2 PPO in CD_2_Cl_2_. The spectrum provides no evidence for the formation of a Ni–F species, which contradicts the proposed formation of the PFPh₂ ligand through this pathway.

Figure S 29. ^31^P{^1^H} NMR (298 K, CD_2_Cl_2_, 162 MHz) spectrum of 1 in CD_2_Cl_2_ after UV-radiation (365 nm). The formation of PF_3_Ph_2_ (–34.1 ppm, 2t, ^1^*J*_PF, eq_ = 973.1 Hz, ^1^*J*_PF, ax_ = 832.4 Hz) and Ph_2_P–PF_2_Ph_2_ (–4.5 ppm, dt, ^1^*J*_PaPb_ = 269.1 Hz, ^2^*J*_PaF_ = 43.3 Hz; –51 ppm, 2t, ^1^*J*_PaPb_ = 269.1 Hz, ^1^*J*_PbF_ = 724.3 Hz) can be detected.

Figure S 30. ^19^F{^1^H} NMR (298 K, CD_2_Cl_2_, 377 MHz) spectrum of 1 in CD_2_Cl_2_ after UV-radiation (365 nm). The formation of PF_3_Ph_2_ (–35.4 ppm, dd, ^1^*J*_PF, ax_ = 832.4 Hz, ^2^*J*_FF_ = 39.5 Hz; –80.6 ppm, dt, ^1^*J*_PF, eq_ = 973.1 Hz, ^2^*J*_FF_ = 39.5 Hz) and Ph_2_P–PF_2_Ph_2_ (–36.2 ppm, dd, ^2^*J*_PaF_ = 43.3 Hz, ^1^*J*_PbF_ = 724.3 Hz).

Figure S 31. ^31^P{^1^H} NMR (298 K, d_8_-toluene, 162 MHz) spectrum of 1 in d_8_-toluene after UV-radiation (365 nm). The formation of Ph_2_PPPh_2_ (–14.9 ppm, s), PF_3_Ph_2_ (–34.1 ppm, 2t, ^1^*J*_PF, eq_ = 973.1 Hz, ^1^*J*_PF, ax_ = 832.4 Hz) and Ph_2_P–PF_2_Ph_2_ (–4.5 ppm, dt, ^1^*J*_PaPb_ = 269.1 Hz, ^2^*J*_PaF_ = 43.3 Hz; –51 ppm, 2t, ^1^*J*_PaPb_ = 269.1 Hz, ^1^*J*_PbF_ = 724.3 Hz) can be detected.

Figure S 32. ^19^F{^1^H} NMR (298 K, d_8_-toluene, 377 MHz) spectrum of 1 in d_8_-toluene after UV-radiation (365 nm). The formation of PF_3_Ph_2_ (–35.5 ppm, dd, ^1^*J*_PF, ax_ = 832.4 Hz, ^2^*J*_FF_ = 39.5 Hz; –80.6 ppm, dt, ^1^*J*_PF, eq_ = 973.1 Hz, ^2^*J*_FF_ = 39.5 Hz) and Ph_2_P–PF_2_Ph_2_ (–36.1 ppm, dd, ^2^*J*_PaF_ = 43.3 Hz, ^1^*J*_PbF_ = 724.3 Hz).

Ligand exchange reactions

Figure S 33. ^31^P{^1^H} NMR (298 K, C_6_D_6_, 162 MHz) spectrum of 1 with BINAP after UV radiation (365 nm). Following compounds can be detected: 1 (trace, 173.1 ppm, m), [Ni(BINAP)(PFPh_2_)_2_] (trace, 170.8 ppm, m; 31.5 ppm, m), [Ni(BINAP)_2_] (trace, 35.4 ppm, m), free BINAP (–15.0 ppm, s), PF_3_Ph_2_ (–34.1 ppm, 2t, ^1^*J*_PF, eq_ = 973.1 Hz, ^1^*J*_PF, ax_ = 832.4 Hz) and Ph_2_P–PF_2_Ph_2_ (–4.5 ppm, dt, ^1^*J*_PaPb_ = 269.1 Hz, ^2^*J*_PaF_ = 43.3 Hz; –51 ppm, 2t, ^1^*J*_PaPb_ = 269.1 Hz, ^1^*J*_PbF_ = 724.3 Hz).

Figure S 34. ^19^F{^1^H} NMR (298 K, C_6_D_6_, 377 MHz) spectrum of 1 with BINAP after UV radiation (365 nm). Following compounds can be detected: 1 (–134.6 ppm, m), [Ni(BINAP)(PFPh_2_)_2_] (trace, –125.6 ppm, m), PF_3_Ph_2_ (–35.4 ppm, dd, ^1^*J*_PF, ax_ = 832.4 Hz, ^2^*J*_FF_ = 39.5 Hz; –80.6 ppm, dt, ^1^*J*_PF, eq_ = 973.1 Hz, ^2^*J*_FF_ = 39.5 Hz) and Ph_2_P–PF_2_Ph_2_ (–36.2 ppm, dd, ^2^*J*_PaF_ = 43.3 Hz, ^1^*J*_PbF_ = 724.3 Hz).

Figure S 35. ^1^H NMR (298 K, C_6_D_6_, 400 MHz) spectrum of 1 with COD after UV radiation (365 nm). Signals can be assigned to 1 (7.16–6.90 ppm) and free COD (5.58 and 2.21 ppm). No indication of coordinated COD can be found.

Figure S 36. ^31^P{^1^H} NMR (298 K, C_6_D_6_, 162 MHz) spectrum of 1 with COD after UV radiation (365 nm). Following compounds can be detected: 1 (trace, 173.1 ppm, m), Ph_2_PPPh_2_ (–14.9 ppm, s), PF_3_Ph_2_ (–34.1 ppm, 2t, ^1^*J*_PF, eq_ = 973.1 Hz, ^1^*J*_PF, ax_ = 832.4 Hz).


Figure S 37. ^19^F{^1^H} NMR (298 K, C_6_D_6_, 377 MHz) spectrum of 1 with COD after UV radiation (365 nm). The following compounds can be detected: 1 (–134.6 ppm, m), other [Ni(PFPh_2_)_x_ species] (trace, –139.6 ppm, m), PF_3_Ph_2_ (–34.2 ppm, dd, ^1^*J*_PF, ax_ = 832.4 Hz, ^2^*J*_FF_ = 39.5 Hz; –80.6 ppm, dt, ^1^*J*_PF, eq_ = 973.1 Hz, ^2^*J*_FF_ = 39.5 Hz), Ph_2_P(=O)F (–73.2 ppm, trace, air contamination assumed, d, ^1^*J*_PF_ = 1020 Hz).

Figure S 38. ^31^P{^1^H} NMR (298 K, C_6_D_6_, 162 MHz) spectrum of 1 with PPh_3_ after UV radiation (365 nm). The following compounds can be detected: 1 (173.1 ppm, m), [Ni(PPh_3_)_x_(PFPh_2_)_y_], (170.0 ppm, m; 29.9 ppm, m), [Ni(PPh_3_)_4_] (24.7 ppm, s), PPh_3_ (–4.8 ppm, br), Ph_2_PPPh_2_ (–14.9 ppm, s) PF_3_Ph_2_ (–34.1 ppm, 2t, ^1^*J*_PF, eq_ = 973.1 Hz, ^1^*J*_PF, ax_ = 832.4 Hz).


Figure S 39. ^19^F{^1^H} NMR (298 K, C_6_D_6_, 377 MHz) spectrum of 1 with PPh_3_ after UV radiation (365 nm). The following compounds can be detected: 1 (–134.6 ppm, m), [Ni(PPh_3_)_x_(PFPh_2_)_y_] (around –134 ppm, m), PF_3_Ph_2_ (–34.2 ppm, dd, ^1^*J*_PF, ax_ = 832.4 Hz, ^2^*J*_FF_ = 39.5 Hz; –80.6 ppm, dt, ^1^*J*_PF, eq_ = 973.1 Hz, ^2^*J*_FF_ = 39.5 Hz) and Ph_2_P–PF_2_Ph_2_ (–38.8 ppm, dd, ^2^*J*_PaF_ = 43.3 Hz, ^1^*J*_PbF_ = 724.3 Hz).

Figure S 40. ^31^P{^1^H} NMR (298 K, C_6_D_6_, 162 MHz) spectrum of 1 with dppf after UV radiation (365 nm). The following compounds can be detected: 1 (173.1 ppm, m), [Ni(dppf)(PFPh_2_)_2_] (164 ppm, m; 21.7 ppm, m), [Ni(dppf)_2_] (24.2 ppm, s), Ph_2_PPPh_2_ (–14.9 ppm, s), free dppf (–15.0 ppm, br), Ph_2_P–PF_2_Ph_2_ (–4.5 ppm, dt, ^1^*J*_PaPb_ = 269.1 Hz, ^2^*J*_PaF_ = 43.3 Hz; –51.4 ppm, 2t, ^1^*J*_PaPb_ = 269.1 Hz, ^1^*J*_PbF_ = 724.3 Hz).

Figure S 41. ^19^F{^1^H} NMR (298 K, C_6_D_6_, 377 MHz) spectrum of 1 with dppf after UV radiation (365 nm). Following compounds can be detected: 1 (–134.6, ppm, m), [Ni(dppf)(PFPh_2_)_2_] (–130.4 ppm, m), PF_3_Ph_2_ (trace, –35.4 ppm, dd, ^1^*J*_PF, ax_ = 832.4 Hz, ^2^*J*_FF_ = 39.5 Hz; –80.6 ppm, dt, ^1^*J*_PF, eq_ = 973.1 Hz, ^2^*J*_FF_ = 39.5 Hz) and Ph_2_P–PF_2_Ph_2_ (–35.5 ppm, dd, ^2^*J*_PaF_ = 43.3 Hz, ^1^*J*_PbF_ = 724.3 Hz).

Figure S 42. ^31^P{^1^H} NMR (298 K, C_6_D_6_, 162 MHz) spectrum of 1 with dppe after heat (100°C). The following compounds can be detected: 1 (173.1 ppm, m), [Ni(dppe)(PFPh_2_)_2_] (171.2 ppm, m; 41.4 ppm, m), Ni(dppe)_2_ (44.1 ppm, s), free Ph_2_PF (163.5 ppm, d, ^1^*J*_PF_ = 884.3 Hz), free dppe (–12.9 ppm, s).

Figure S 43. ^19^F{^1^H} NMR (298 K, C_6_D_6_, 377 MHz) spectrum of 1 with dppe after heat (100°C). Following compounds can be detected: 1 (–134.6 pp, m), [Ni(dppe)(PFPh_2_)_2_] (–128.6 ppm, m), PFPh_2_ (–195.4 ppm, d, ^1^*J*_PF_ = 884.3 Hz).

Figure S 44. Tautomeric equilibria of Ph_2_P(=O)–PPh_2_ and Ph_2_P(=O)–P(=O)Ph_2_ and formal oxidation numbers given in red.

# Further characterisation of compound 1


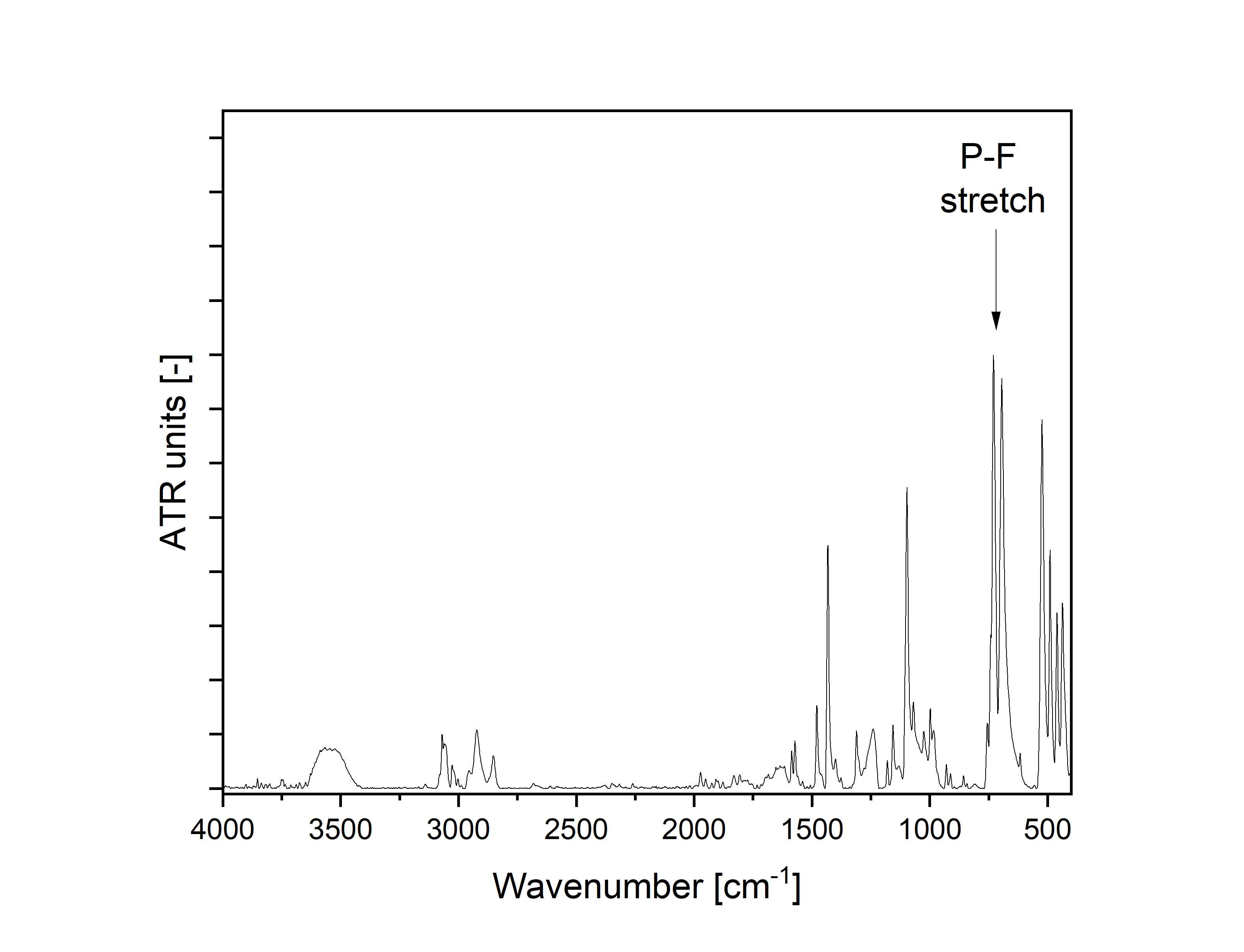


Figure S 45. ATR-IR spectrum of 1 recorded at 298 K under a nitrogen atmosphere.

Figure S 46. Simulated (top) and experimental (bottom) powder XRD pattern of **1**.





Figure S 47. EPR spectrum of 1. Microwave Power = 2 mW, frequency = 9.430734 MHz (X-Band), conversion time = 1.28 ms.


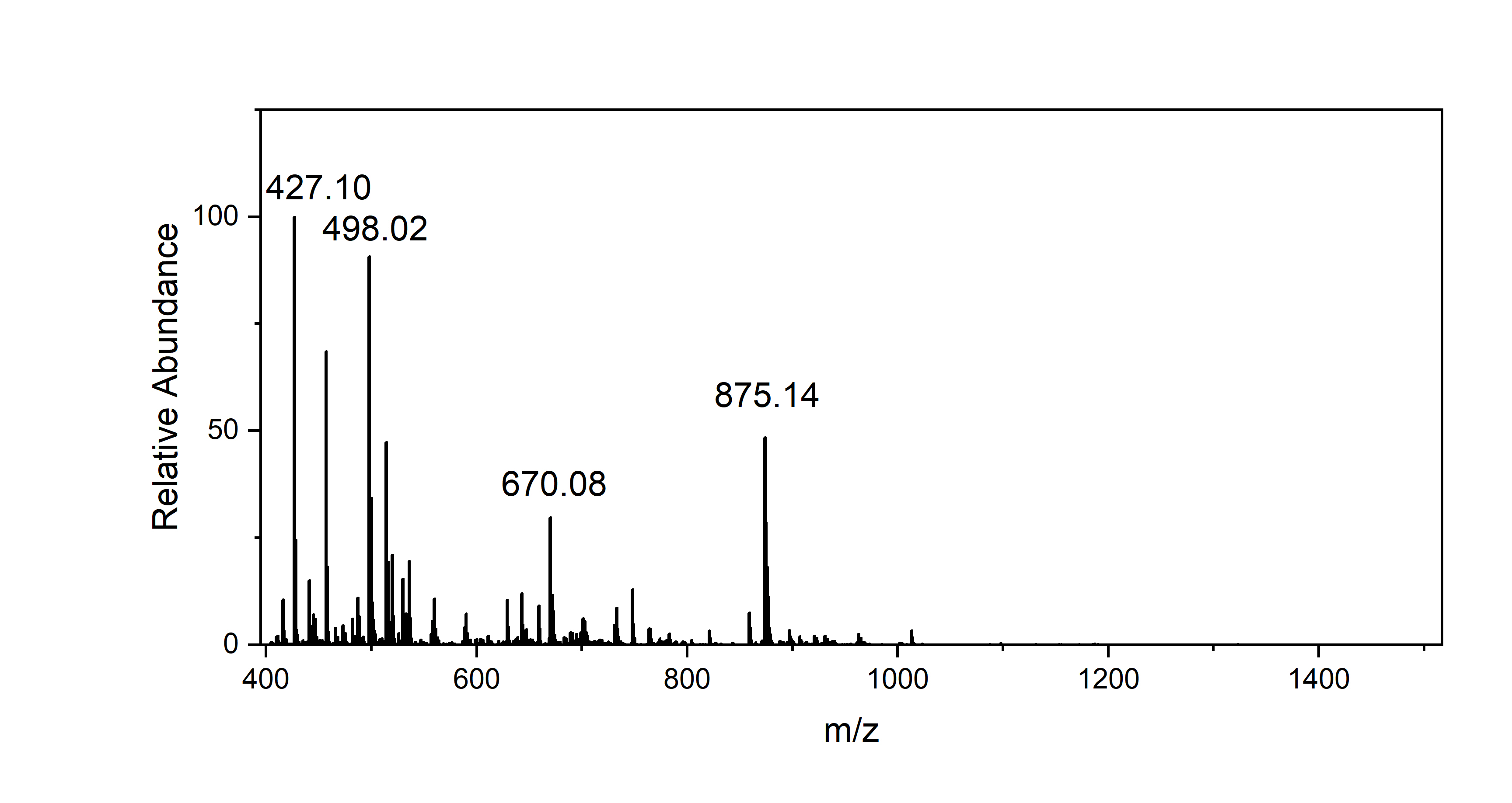


Figure S 48. ESI-MS spectrum of 1 in dcm (positive mode). m/z([Ni(PFPh_2_)_4_] + H)^+^ = 875.14


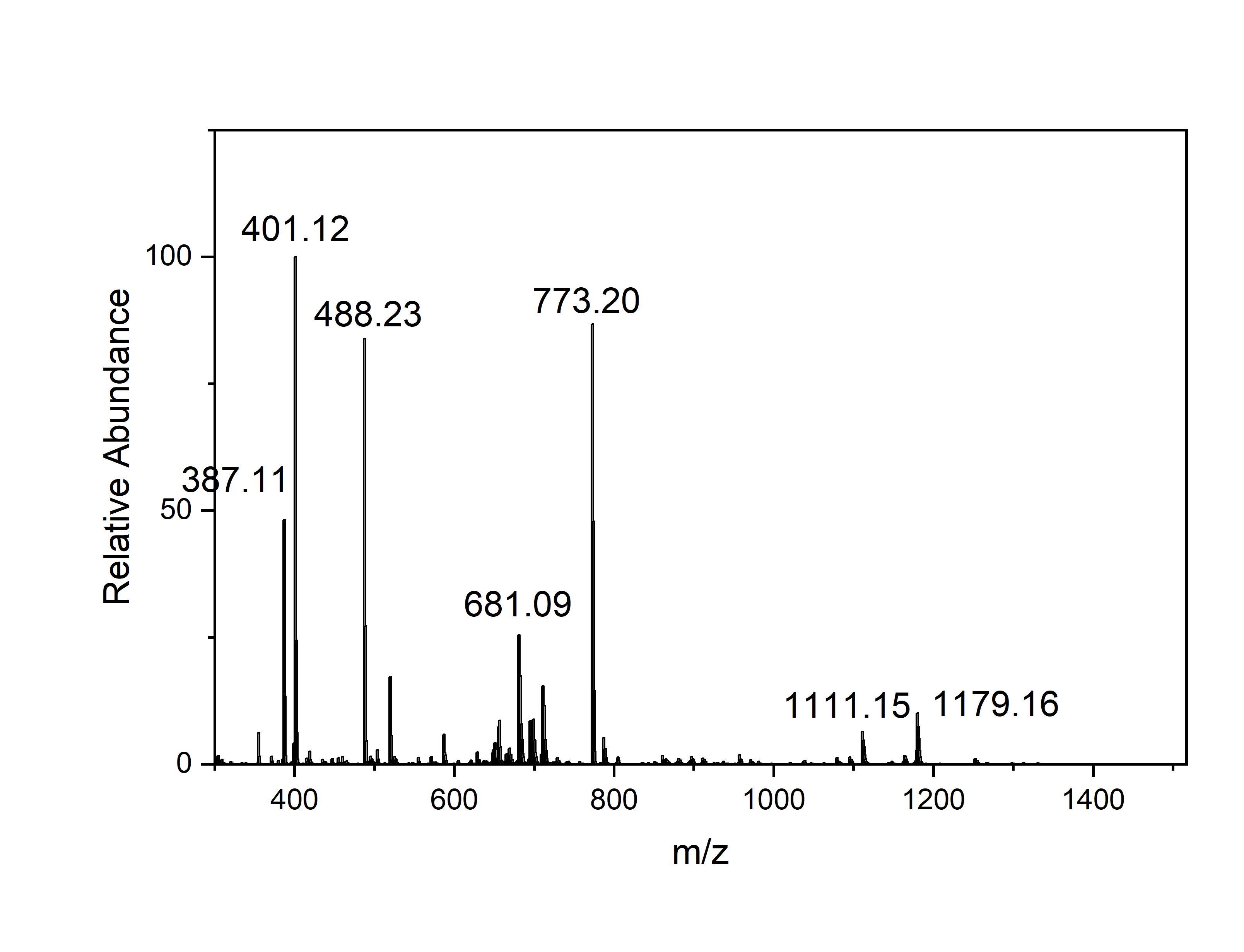


Figure S 49. ESI-MS spectrum of the reaction solution for the formation of 1 in dcm (positive mode). m/z(Ph_2_P(=O)PPh_2_ + H)^+^ = 387.11; m/z(2 Ph_2_P(=O)PPh_2_ + H)^+^ = 773.20.


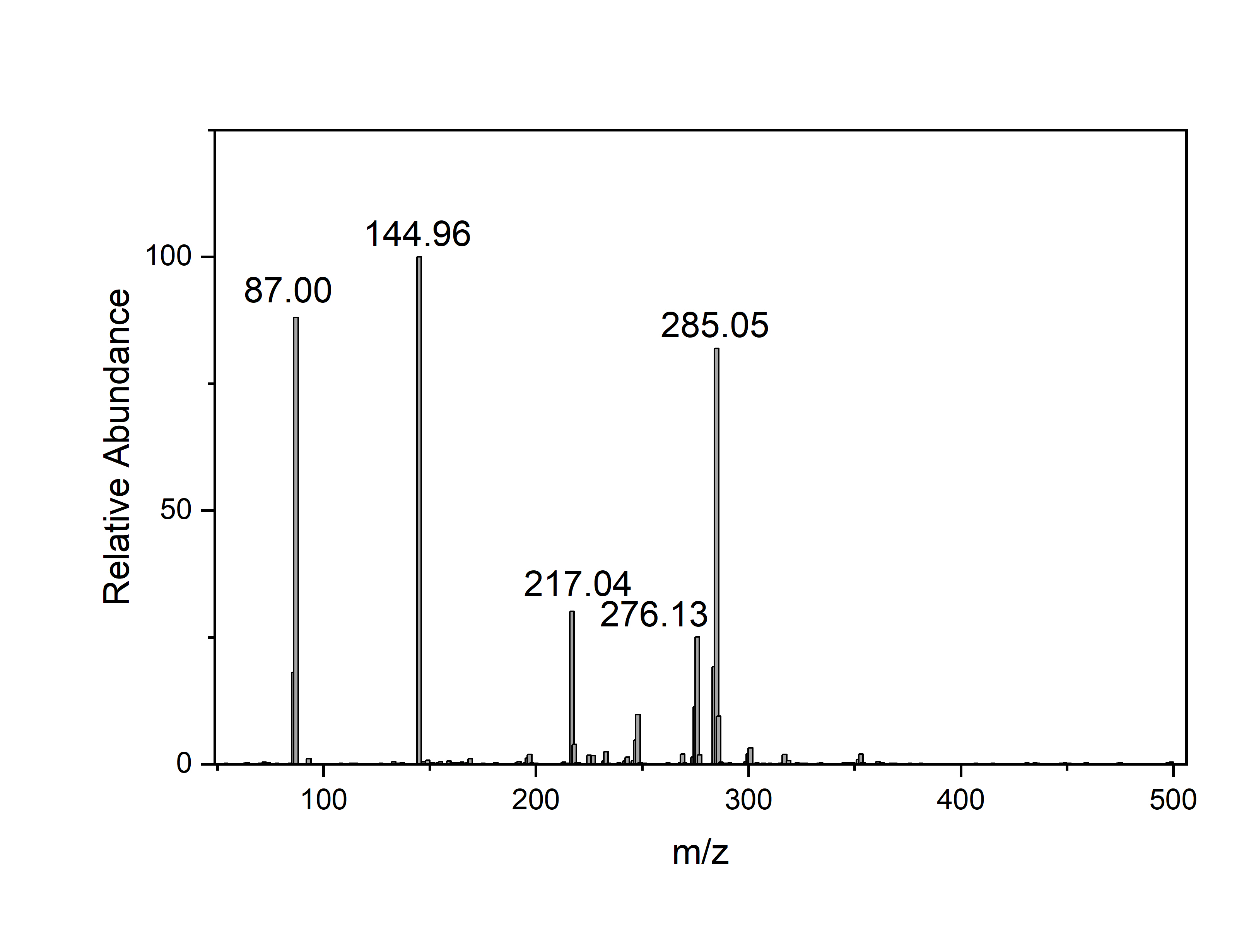


Figure S 50. ESI-MS spectrum of the reaction solution for the formation of 1 in dcm (negative mode). m/z(Ph_2_POO^‑^) = 217.04; m/z(BF_4_^–^) = 87.00.


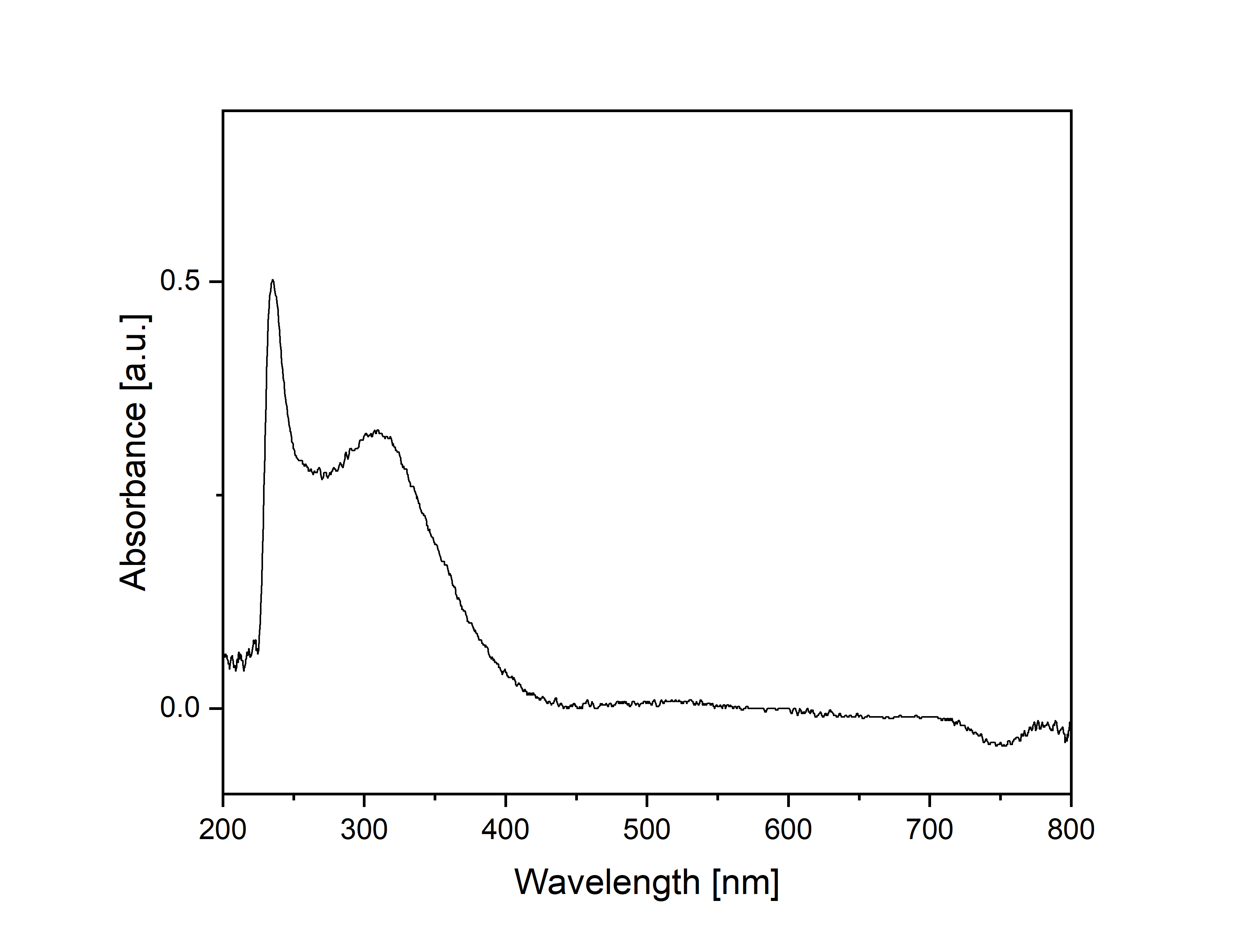


Figure S 51. UV/Vis spectrum of **1** in DCM solution (5 · 10^-5^ mol L^-1^).


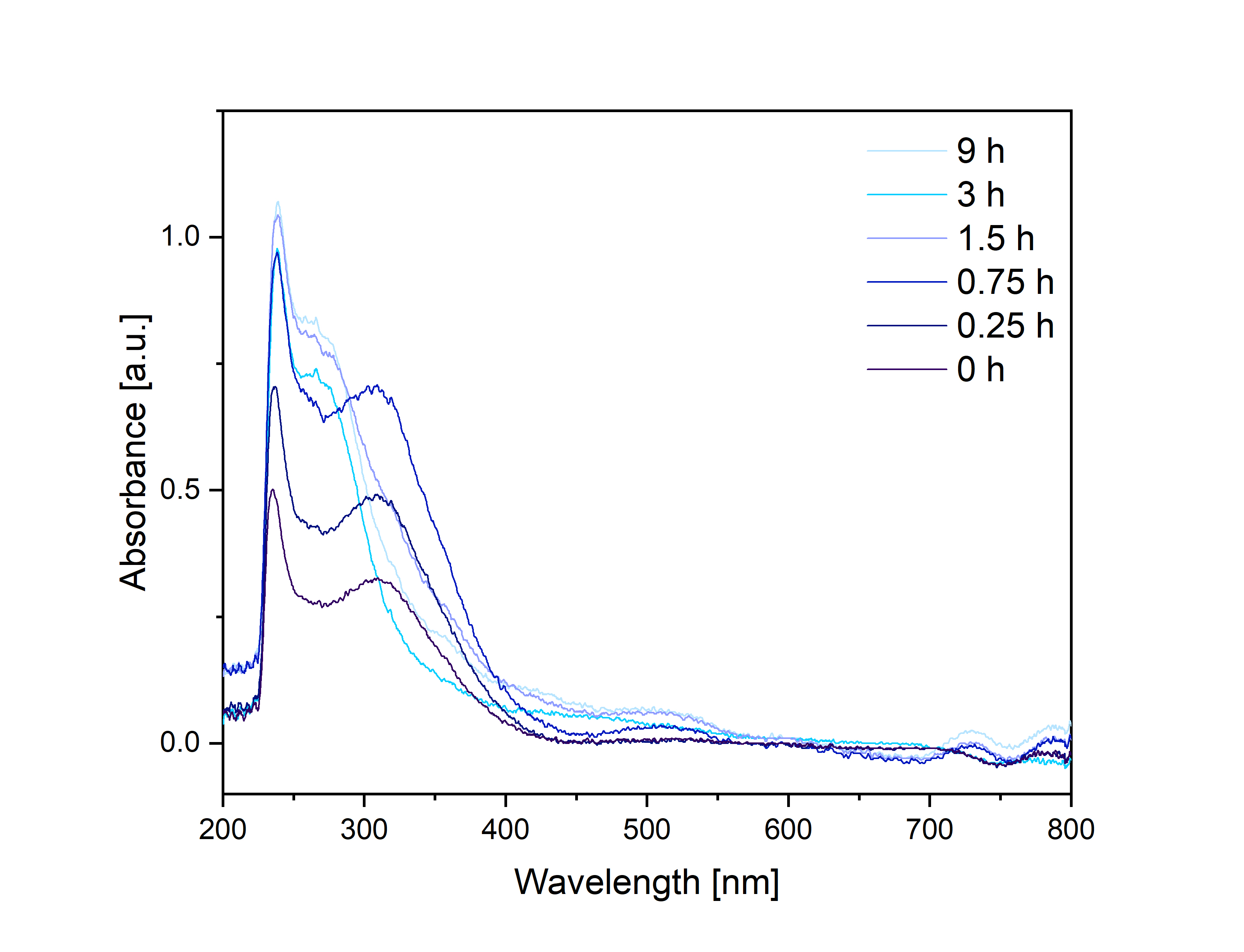


Figure S 52. UV/Vis spectrum of **1** in DCM solution (5 · 10^-5^ mol L^-1^) and decomposition of **1** under UV radiation over time.

# X-ray Crystallographic details

Single crystals were measured on a STOE STADIVARI and on a STOE METAL JET D2 diffractometer. The STOE STADIVARI is equipped with an open Eulerian cradle (4-circle) and a DECTRIS PILATUS pixel detector at 100 K with a microfocus molybdenum source (Mo-Kα, λ = 0.71073 Å) using a graphite monochromator as radiation source. The STOE METAL JET 2D contains a EIGER4M Detector and a graded multilayer mirror monochromator. A liquid-gallium-jet anode is utilized as radiation source. The data reduction was conducted with X-Area version 1.73.1.0 (STOE, 2018)^[8]^ using the semi-empirical absorption correction by X-RED with scaling of the reflection intensities by LANA included in X-Area. Structures were solved by means of dual space methods with SHELXT-2015^[9]^ and refinement was performed with SHELXL-2019^[10]^ using the WinGX^[11]^ program suite. Full-matrix least-square routines against F^2^ were carried out. Hydrogen atoms were calculated on idealized positions. Pictures were generated with the program DIAMOND^[12]^. For these thermal ellipsoids are shown with 30% probability and hydrogen atoms as well as co-crystallised solvent molecules are omitted for clarity. CCDC 2379067 (**1**), CCDC 2379068 (**2**) and CCDC 2379069 (**3)** contain the supplementary crystallographic data for this paper. These data can and additional information can be obtained free of charge via https://summary.ccdc.cam.ac.uk/structure-summary-form (or from the Cambridge Crystallographic Data Centre, 12 Union Road, Cambridge CB2 1EZ, UK; fax: (+44)1223-336-033; or [deposit@ccdc.cam.uk](mailto:deposit@ccdc.cam.uk)).

PXRD measurements were conducted in a glass capillary on a Stoe STADI-MP diffractometer operating with a Ge-mono-chromatized Cu-Kα (λ = 1.54178 Å) radiation in transmission mode. The theoretical powder pattern from the single crystal structures was calculated using MERCURY.

Comments on compound **3**:

We suggest that compound **3** can be identified as [Ni(OPOPh_2_)_6_(BF)_2_] instead of the reported [Ni(OP(H)Ph_2_)_6_(BF_4_)_2_]^[13]^ meaning that no hydrogen atoms are directly bound to the phosphorus atom and furthermore that the atom type assigned of the atom located between the P and B atom is oxygen instead of fluorine. This can be explained firstly by the tetrahedral coordination geometry around the P atoms. If hydrogen atoms were present, a different geometry would be expected. From this follows that for a correct charge balance that atom type erroneously assigned to fluorine has to be oxygen. Otherwise, the nickel atom would have to have a formal charge of +8 to balance the deprotonated Ph_2_PO^–^ ligands and the counter ion which is highly unlikely. In compound **3**, the negative charge of the six Ph_2_POO^–^ ligands can be balanced by two BF_2_^+^ units and nickel in the oxidation state +2. The presence of **3** instead of [Ni(OP(H)Ph_2_)_6_(BF_4_)_2_] can also be confirmed by the Hirsfeld test since the difference of the anisotropic displacement parameters of the respective atoms is drastically increased if the O(2) atom is replaced by a F atom.

To summarise, the following factors let us realise the mis-assignment made in reference ^[13]^:

- Mis-assigning F (F1 in Gushwas refined model) instead of O (O2 in our correct model) leads to a too large Hirshfeld difference for the included bonds to the phosphorus and the boron atoms. This is usually a strong indication for a wrong atom type assignment (see ^[14]^)
- If there was truly a hydrogen atom attached to the phosphorus, a different geometry would be expected for it. The phosphorus has a clear tetrahedral coordination environment. If there were a fifth bonded atom, there should at least be strong deformations if not a clearly different environment such as trigonal bipyramidal. Furthermore, this is confirmed by non-^1^H-decoupled ^31^P NMR spectroscopy, which would have shown a strong J-coupling in case of a presence of hydrogen atoms connected the to the phosphorus atoms. Gushwas work lacks this analysis.

Table S 2. Summary of crystallographic data of compounds 1-3.

| Compound | 1 | 2 | 3 |
| --- | --- | --- | --- |
| CCDC | 2379067 | 2379068 | 2379069 |
| Formula | C_48_H_40_P_4_NiF_4_ | C_24_H_20_B_2_F_4_O_4_P_2_ | C_72_H_60_B_2_F_2_NiO_12_P_6_ |
| Formula weight | 875.39 | 531.96 | 1421.35 |
| Crystal system | Tetragonal | Monoclinic | Trigonal |
| Space group | *I*4_1_/*a* | *R*$\bar{3}$*c* | *C*2/*c* |
| a [Å] | 19.0448(7) | 18.0250(7) | 13.0353(8) |
| b [Å] | 19.0448(7) | 8.8487(4) | 13.0353(8) |
| c [Å] | 11.2888(4) | 16.7535(8) | 68.555(7) |
| α [°] | 90 | 90 | 90 |
| β [°] | 90 | 116.271(3) | 90 |
| γ [°] | 90 | 90 | 120 |
| V [Å^3^] | 4094.5(3) | 2396.14(19) | 10088.1(16) |
| Z | 4 | 4 | 6 |
| Z’ | 0.25 | 0.5 | 0.16667 |
| Radiation type | Ga-Kα | Ga-Kα | Mo-Kα |
| Temp. [K] | 150(2) | 180(2) | 100(2) |
| ρ_(calcd)_ [g·cm^–3^] | 1.420 | 1.475 | 1.404 |
| μ [mm^-1^] | 3.841 | 1.436 | 0.500 |
| F(000) | 1808.0 | 1088.0 | 4404 |
| θ range [°] | 7.92-124.964 | 9.522-125.028 | 3.564-57.616 |
| Limiting indices | -25 ≤ h ≤ 22,  -23 ≤ k ≤ 21  -5 ≤ l ≤ 14 | -23 ≤ h ≤ 16  -11 ≤ k ≤ 11  -22 ≤ l ≤ 21 | -17 ≤ h ≤ 14  -16 ≤ k ≤ 15  -90 ≤ l ≤ 89 |
| Reflections collected/unique^a^ | 5965 / 2428 [R(int) = 0.0143] | 13369 / 2828  [R(int) = 0.0178] | 13371 / 2716  [R(int) = 0.1426] |
| Data/restraints/param | 2428/0/129 | 2828/0/163 | 2716/0/144 |
| Completeness to (θ) | 98.1% (53.597°) | 97.1% (53.597°) | 100.0% (25.242°) |
| Final R indices (I > 2σ(I))^b^ | R_1_ = 0.0371, wR_2_ = 0.0889 | R_1_ = 0.0295, wR_2_ = 0.0847 | R_1_ = 0.0451, wR_2_ = 0.1147 |
| R indices (all data) | R_1_ = 0.0382, wR_2_ = 0.0895 | R_1_ = 0.0310, wR_2_ = 0.0857 | R_1_ = 0.0597, wR_2_ = 0.1176 |
| Absolute Structure Parameter | n/a | n/a | n/a |
| Goodness  of fit^c^ on F^2^ | 1.042 | 1.089 | 0.992 |
| Largest diff. peak and hole [Å*^−^*^3^] | 1.18/-0.38 | 0.31/-0.35 | 0.81/-0.58 |

^a^ R_int_ = Σ|F_o_^2^ − F_o_^2^(mean)|/ΣF_o_^2^, ^b^ R_1_ = Σ||F_o_| − |F_c_||/Σ|F_o_|, wR_2_ = {Σ[*w*(F_o_^2^ − F_c_^2^)^2^]/Σ[*w*(F_o_^2^)^2^]}^1/2^, ^c^ GooF = {S/(*n −p*)}^1/2^ = {Σ[*w*(F_o_^2^ − F_c_^2^)^2^]/(*n − p*)}^1/2^.


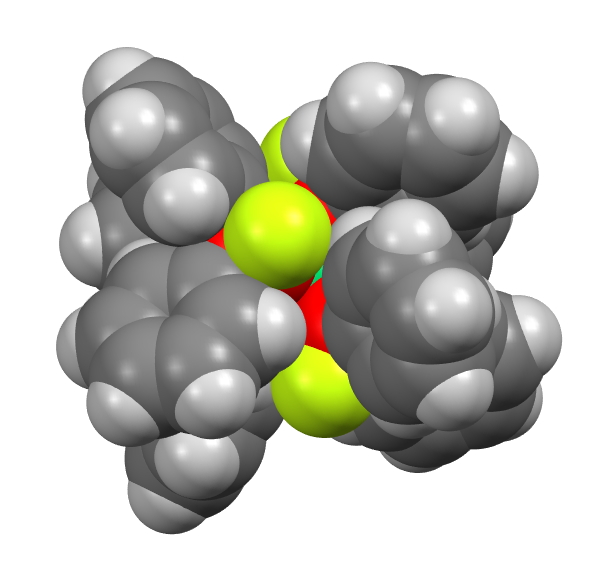


Figure S 53. Space filling diagram of **1**.

# Comparison with reported P–F containing compounds

Table S 3. Literature comparison of P–F and Ni–P bond lengths as well as NMR signals in pure fluorophosphine ligands, fluorophosphine-stabilized nickel complexes and fluorodiphenyl phosphine complexes.

| Compound | P–F bond [Å] | Ni–P bond [Å] | ^31^P δ [ppm] | ^19^F δ [ppm] | *J*_PF_ [Hz] | Literature |
| --- | --- | --- | --- | --- | --- | --- |
| PF_3_ | 1.569(1)^[b]^ | - | 103.9 | –33.5 | 1403 | ^[15]^ |
| Bu*^t^*PF_2_ | 1.589(4) | - | 231.2 | 111.5 | 1219 | ^[16]^ |
| (Bu*^t^*)_2_PF | 1.619(7) | - | 214.9 | –216.7 | 865 | ^[16a, 17]^ |
| PhPF_2_ | - | - | 206.4 | –91.2 | 1166 | ^[18]^ |
| Ph_2_PF | - | - | 162 | –195 | 883 | ^[18-19]^ |
| (CF_3_)_2_PF | - | - | –124.6 | 221.6 (PF) | 1002 | ^[20]^ |
|  | 1.616(4) | - | 220.8 | –94.39 | 918 | ^[21]^ |
|  | 1.6544(14) | - | 111.2 | –33.6 | 1066 | ^[22]^ |
|  | 1.6569(15) | - | 150.1 | –81.0 | 1035 | ^[23]^ |
|   = CgPF | 1.574(7) | - | 217.3 | –207.9 | 808 | ^[17]^ |
| [Ni(PFPh_2_)_4_] = **1** | 1.6465(12) | 2.1296(4) | 173.1 | –134.5 | 850.25 | This work |
| [Ni(PF(CF_3_)_2_)] | - | - | - | 158.95 | 998 | ^[24]^ |
| [Ni(PF_2_(CF_3_))] | - | - | - | 60.59 | 1190 | ^[24]^ |
| [Ni(COD)(CgPF)_3_] | - | - | 164.5; 163.0 | - | 821;  839^[c]^ | ^[17]^ |
| [NiBr_2_(PF*^t^*Bu_2_)_2_]] | 1.579(7) | 2.232(3) | Hardly soluble | 175 (broad) | Broad duplet | ^[25]^ |
|  | 1.6189(10) | 2.1167(4)^[d]^ | 151.5 | –20.7 | 1160 | ^[26]^ |
|  | 1.614(2) | - | 181.4 | –110.7 | 856 | ^[27]^ |
|  | 1.616(2) |  | 188.8 | –109.7 | 848 | ^[27a]^ |
|  | 1.609(1) | - | - | - | - | ^[27a]^ |
|  | 1.614(2) | - | 201.3 | 139.7 | 867 | ^[28]^ |
|  | 1.641(15)–1.645(13) | - | 216.4 | - | 903 | ^[29]^ |

^[a]^ Taken from ^19^F{^1^H} spectra ^[b]^ Structure studied by gas electron diffraction ^[c]^ NMR of the reaction solution ^[d]^ Ni– P bond length and ^31^P{^1^H} signal of blue marked atoms.

# Catalytic tests

Initial studies were conducted with iodobenzene and phenylboronic acid using K_2_CO_3_ as base. No conversions with **1** at room temperature (with and without UV radiation) have been observed (Table 1 manuscript, entries 1-2). After a well-adjusted activation procedure, based on UV radiation (365 nm) for 15 min followed by heating to 100°C, a conversion of 72% could be achieved. However, as shown before as part of the stability studies, complex **1** decomposes during UV light radiation. To overcome this stability issue, while employing the catalytic potential of **1**, PPh_3_, dppe and dppf were added as additional ligands (Table 1 manuscript, entries 5-11). Again, the reaction mixtures were exposed to UV radiation (365 nm) for 15 min and subsequently heated to 100°C to guarantee sufficient ligand exchange. Surprisingly, the addition of PPh_3_ and dppe leads to a drop of conversion (Table 1 manuscript, entries 5 and 7) which might stem from too stable complexes with these ligands which block active sites. Since the ligand exchange with dppe occurred already at elevated temperatures (100°C), the reaction was repeated without UV radiation (Table 1 manuscript, entry 6). However, no significant difference in conversion can be witnessed by adopting the activation procedure. Subsequently, coupling reactions with dppf were conducted using different activation procedures. Contrary to dppe, the additional application of UV light leads to an increase in conversion from 80% to 100%. Consequently, **1** can act as pre-catalyst in the presence of dppf with the mixed-ligand system showing a great potential as catalyst in Suzuki-Miyaura coupling reactions.

Table S 4. Suzuki-Miyaura couplings of iodobenzene (1 eq.) and phenylboronic acid (1.5 eq.). Reaction conditions, if not noted otherwise: 2.5 mol% catalyst, 5 mol% ligand, 3 equivalents K_2_CO_3_, toluene as solvent and *n*-decane as internal standard. Catalytic tests were conducted at room temperature (r.t.; without UV radiation), at r.t. after 15 min of exposure to UV light (365 nm), at 100°C (without UV radiation) or at 100°C after 15 min of exposure to UV light (365 nm). Total reaction times were 14 h. Conversions of arylhalides were determined using GC-MS. Product formation occurs with 100% selectivity.

| Entry | Ni(0) source | Ligand | Conditions | Conversion [%] |
| --- | --- | --- | --- | --- |
| 1 | - | dppf | r.t. | 0 |
| 2 | - | dppf | UV, r.t. | 0 |
| 3 | - | dppf | 100°C | 0 |
| 4 | - | dppf | UV, 100°C | 0 |
| 5 | 1^[a]^ | dppf | UV^[a]^, 100°C | 0 |
| 6 | [Ni(COD)_2_] | dppf | r.t. | 0 |
| 7 | [Ni(COD)_2_] | dppf | UV, r.t. | 0 |
| 8 | [Ni(COD)_2_] | - | 100°C | 61 |

[a] without addition of K_2_CO_3_


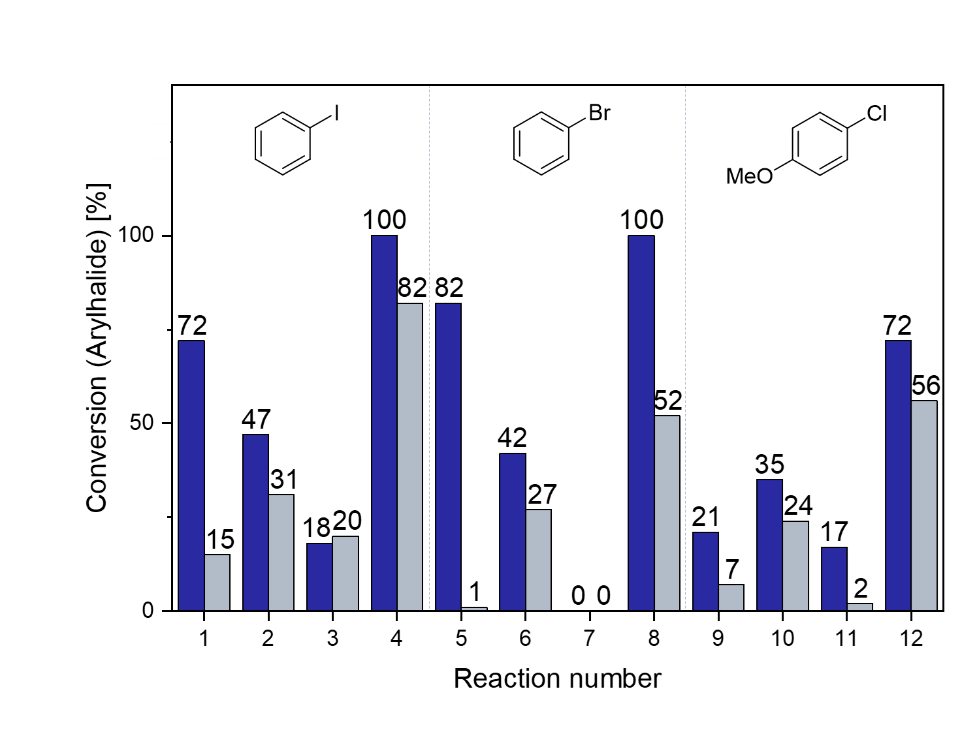


Figure S 54. Comparison of arylhalide conversion of 1 (blue) vs. [Ni(COD)_2_] (grey) in the Suzuki-Miyaura coupling of phenylboronic acid with iodobenzene (reactions 1–4), bromobenzene (reactions 5–8) and 4-chloroanisole (reactions 9–12). Either no ligand (1, 5, 9), PPh_3_ (2, 6, 10), dppe (3, 7, 11) or dppf (4, 8, 12) was added. Reaction conditions: 2.5 mol% catalyst, 5 mol% ligand, 3 equivalents K_2_CO_3_, toluene as solvent and *n*-decane as internal standard. Samples were exposed to UV light (365 nm) and heated to 100°C for 14 h. Conversions of arylhalides were determined *via* GC-MS.

Table S 5. Comparison of arylhalide conversion and selectivity of 1 vs. [Ni(COD)_2_] in the Suzuki-Miyaura coupling of phenylboronic acid with iodobenzene, bromobenzene and 4-chloroanisole. Either no ligand (1, 5, 9), PPh_3_ (2, 6, 10), dppe (3, 7, 11) or dppf (4, 8, 12) was added. Reaction conditions: 2.5 mol% catalyst, 5 mol% ligand, 3 equivalents K_2_CO_3_, toluene as solvent and *n*-decane as internal standard. Samples were exposed to UV light (365 nm) and heated to 100°C for 14 h. Conversions of arylhalides were determined *via* GC-MS.

| Entry | Ni(0)  source | Ligand | Conv. [%] I-benzene | Select. [%]  product | Conv. [%] Br-benzene | Select. [%]  product | Conv. [%]  Cl-anisole | Select. [%]  product |
| --- | --- | --- | --- | --- | --- | --- | --- | --- |
| 1 | 1 | - | 72 | 100 | 82 | 100 | 21 | 63 |
| 2 | 1 | PPh_3_ | 47 | 100 | 42 | 100 | 35 | 92 |
| 3 | 1 | dppe | 18 | 100 | 0 | 0 | 0 | 0 |
| 4 | 1 | dppf | 100 | 100 | 100 | 100 | 72 | 95 |
| 5 | [Ni(COD)_2_] | - | 15 | 100 | 0 | 0 | 0 | 0 |
| 6 | [Ni(COD)_2_] | PPh_3_ | 31 | 100 | 27 | 100 | 24 | 62 |
| 7 | [Ni(COD)_2_] | dppe | 20 | 100 | 0 | 0 | 0 | 0 |
| 8 | [Ni(COD)_2_] | dppf | 82 | 100 | 52 | 100 | 56 | 99 |
| 9 | - | - | 0 | 0 | 0 | 0 | 0 | 0 |


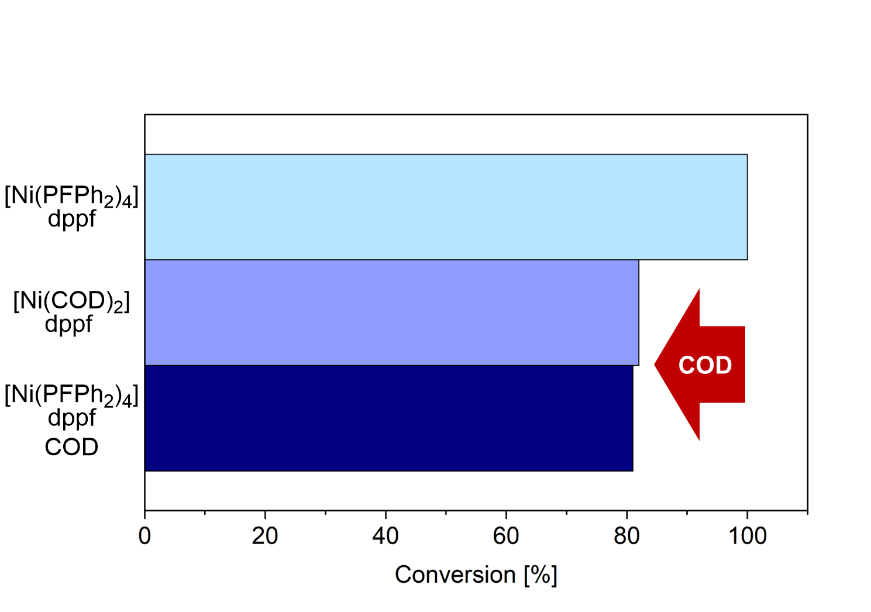


Figure S 55. Superior behaviour of 1 vs. [Ni(COD)_2_]: with usage of 1 a significant increase in conversion can be achieved which might be due to a lower number of blocked active sites, as it is the case if COD is present in the reaction mixture.


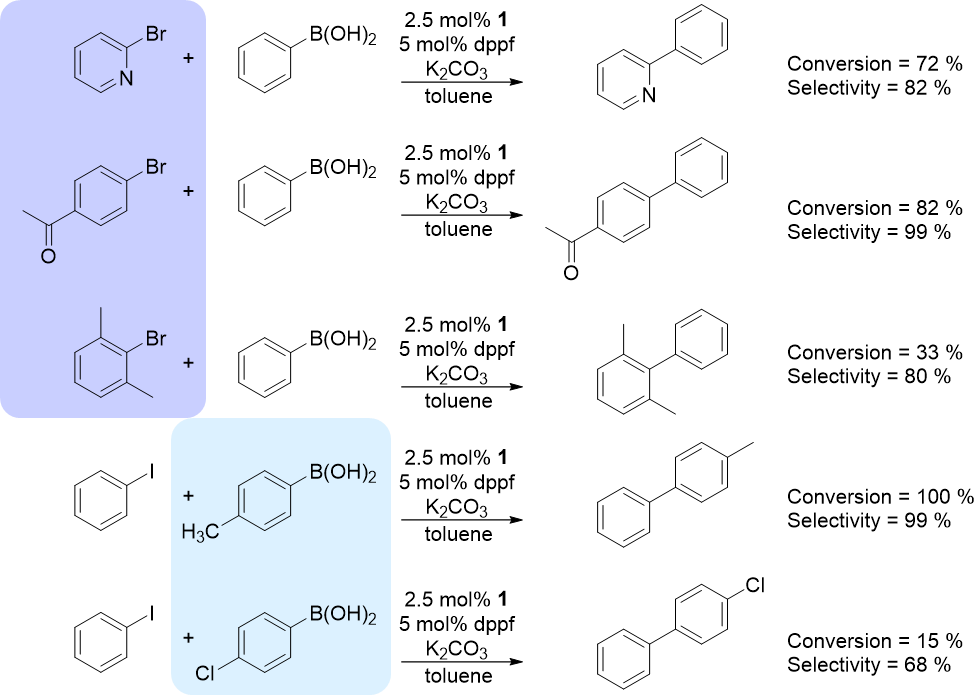


Figure S 56. Substrate scope and respective conversions and selectivities of 1 using different substrates for Suzuki-Miyaura couplings.

# Poisoning experiments

**Dct poisoning**

A first poisoning experiment was conducted using dct. Dct is known to poison homogeneous catalysts selectively, while heterogeneous catalysts are in most cases not interacting with this compound.^[30]^ However, it is important to mention that poisoning experiments with dct have been mainly investigated in the context of 4d and 5d metal-based catalysts, whereas 3d metals are assumed to form more labile complexes with dct resulting in a less efficient poisoning effect.^[31]^ In this study, the addition of dct impacts the catalytic performance of **1** in combination with dppf after UV and temperature activation. However, no sufficient poisoning can be achieved even with 4 equivalents of added poison. Hereby, a significant dependence of the timing of the poison addition was observed, since stirring of the catalyst with the poison 1 h prior to the reaction results in a more severe poisoning effect. If the poison is added after 3 hours of the reaction also a tremendous drop of the conversion can be observed. However, some non-poisoned species remain in the reaction mixture, since iodobenzene is further converted. This is in accordance with earlier studies, in which despite the addition of dct, high conversions can be achieved in Suzuki-Miyaura couplings.^[32]^ For heterogeneous catalysts no effect on the catalyst activity could be observed if dct was added.^[31, 33]^

**Maitlis filtration test**

For the Maitlis filtration test, the reaction mixture was filtered over celite after the reaction proceeded for 2.5 h (Table S 7). One control experiment was conducted without filtration for the same reaction time (Table S 7, entry 1). The filter column was washed with toluene and the filtrate as well as the filter aid have been separately used for further catalytic testing. Phenylboronic acid, iodobenzene and base were added to the filter aid approach, which was resuspended in toluene. The filtrate was used as such, except addition of half of the initial amount of base, to compensate any base loss during the filtration due to insufficient solubility. After an overall reaction time of 8 hours, including the period before filtration, the conversion in the filtrate was determined *via* GC-MS.

Additionally, the catalytic performance of the filter aid was analysed after 8 hours indicating a drop in conversion to 28% which again underlines the homogeneous nature of **1** together with dppf after UV and heat activation. Hereby it is assumed that the residual conversion can be attributed to precipitation of the catalytically active species during the filtration due to the temperature drop as well as insufficient washing of the column.

Table S 6. Poisoning experiments of Suzuki-Miyaura couplings of iodobenzene (1 eq.) and phenylboronic acid (1.5 eq.) using different amounts of dct or CS_2_. Reaction conditions, if not noted otherwise: 2.5 mol% catalyst, 5 mol% ligand, 3 equivalents K_2_CO_3_, toluene as solvent and *n*-decane as internal standard. Catalysis was conducted at 100°C after 15 min of exposure to UV light (365 nm). Total reaction times were 14 h if not noted otherwise. Conversions of arylhalides were determined using GC-MS. Product formation occurs with 100% selectivity.

| Entry | Poison | Reaction time [h] | Eq. poison related to 1 | Conversion [%] |
| --- | --- | --- | --- | --- |
| 1 | - | 2.5 | - | 29 |
| 2 | - | 14 | - | 100 |
| 3 | dct | 14 | 0.5 | 100 |
| 4 | dct | 14 | 1.0 | 75 |
| 5 | dct | 14 | 2.0 | 59 |
| 6 | dct | 14 | 4.0 | 35 |
| 7 | dct | 14^[a]^ | 2.0 | 56 |
| 8 | dct | 14^[b]^ | 2.0 | 43 |
| 9 | CS_2_ | 14 | 0.25 | 29 |
| 10 | CS_2_ | 14 | 2.0 | 15 |

^[a]^ addition of dct after 2.5 h of reaction time ^[b]^ addition of dct to 1, 1 h prior to start of the reaction.

Table S 7. Filtration test of Suzuki-Miyaura couplings of iodobenzene (1 eq.) and phenylboronic acid (1.5 eq.). Reaction conditions, if not noted otherwise: 2.5 mol% catalyst, 5 mol% ligand, 3 equivalents K_2_CO_3_, toluene as solvent and *n*-decane as internal standard. Catalysis was conducted at 100°C after 15 min of exposure to UV light (365 nm). Total reaction times were 8 h. Filtration was conducted after 2.5 h and filtrate and filter aid were treated separately afterwards. Conversions of arylhalides were determined using GC-MS. Product formation occurs with 100% selectivity.

| Entry | Description | Conversion [%] |
| --- | --- | --- |
| 1 | No filtration | 64 |
| 2 | Filtrate^[a]^ | 61 |
| 3 | Filter aid^[b]^ | 28 |

^[a]^ after filtration, 20 mg of K_2_CO_3_ are added to compensate base loss during filtration ^[b]^ after filtration filter aid was recollected and used as catalytic material for conversion of fresh substrate.

# Computational details

All calculations, except the NBO analyses, were carried out using the ORCA package in the gas phase.^[34]^ For all ORCA calculations, atom-pairwise dispersion corrections with the Becke-Johnson damping scheme (D3BJ) were utilised.^[35]^ Density fitting techniques, also called resolution-of-identity approximation (RI), were used for GGA calculations, whereas the RIJCOSX^[36]^ approximation was used for hybrid calculations. Geometry optimisations and frequency analyses, to confirm the absence of any imaginary frequencies, were carried out at the B3LYP^[37]^/def2-TZVP^[38]^ level of theory. The geometry optimisation of **1** is based on the coordinates obtained from the crystal structure. The therewith obtained structure is assigned a *C*_1_ symmetry. However, by using the UseSym keyword it was possible to detect the point group *S*_4_ with a threshold of 1.0e^-1^. The B3LYP functional was selected since it represents the experimental IR spectrum of **1**, especially with respect to the P–F stretching frequency, best. As a reference, the TPSS^[39]^ functional was utilised. Charge distributions of various Ni(0) complexes were examined (after a successful geometry optimisation and frequency calculation) employing CHELPG charges (charges from electrostatic potentials using a grid-based method) developed by C. M. Breneman and K. B. Wiberg^[40]^ using the ORCA package at the B3LYP^[37]^/def2-TZVP^[38]^ level of theory. In addition, NBO/NPA analyses^[41]^ were computed using the NBO 6.0 package to further examine the charge distribution.^[42]^

The computational investigation of phosphine donor properties is increasingly becoming the method of choice, as the experimental determination through isolation and spectroscopic analysis of the corresponding metal carbonyl complexes is challenging, due to the ability of many phosphines to adopt various coordination modes.^[43]^ For the determination of the Tolman electronic parameter, geometry optimisation and subsequent frequency analyses (to identify the symmetric A_1_ CO stretch) of various [Ni(CO)_3_L] complexes using B3LYP^[37]^/def2-TZVP^[38]^ were carried out. Time-dependent density functional theory (TD-DFT)^[44]^ calculations at the B3LYP^[37]^/def2-TZVP^[38]^ level of theory were utilised to investigate the decomposition reaction of **1** under UV light. In this context, also solvent effects were considered by using a conductor-like polarizable continuum model (CPCM) with a refractive index of 1.424 and the dielectric constant of 9.08 (dichloromethane). For plotting the spectra, the orca_mapspc programme was used with a 10 cm^-1^ broadening for the IR spectra and a 2500 cm^-1^ broadening for the UV/Vis spectra.

Table S 8. Comparison of the calculated structure in *C*_1_ symmetry with the structure in *S*_4_ geometry, obtained from a symmetrisation, as well as the experimentally determined crystal structure. The comparison shows almost identical bond lengths and angles. Level of theory: B3LYP, def2-TZV.

| **Selected bond lengths and angles** | **Calculated structure**  **Symmetry: *C*_1_** | **Structure-corrected structure**  **Symmetry: *S*_4_** | **Crystal structure** |
| --- | --- | --- | --- |
| Ni–P | 2.1454-2.1455 Å | 2.1454 Å | 2.1296 Å |
| P–F | 1.605 Å | 1.6305 Å | 1.647 Å |
| P–Ni–P | 108.23°, 111.97° | 108.24°, 111,97° | 108.73, 110.96° |
| F–P–Ni | 115.92-115.93° | 115.93° | 114.98 |

Figure S 57. Calculated IR spectra of **1**. Level of theory: TPSS and B3LYP, def2-TZV. For all following calculations B3LYP was the functional of choice since it represents the experimental IR spectrum very well (P–F calc. using B3LYP 735 cm^-1^ versus P–F exp. 730 cm^-1^). When assuming a *S*_4_ symmetry of **1**, there should be three IR-active P–F stretching modes being present, namely one A and two degenerate E stretching modes. The double degenerate E band, which is an asymmetric stretching vibration, becomes obvious at 735 cm^-1^ in the calculations at the B3LYP/def2TZVP level of theory. The A band, which is a symmetric stretching vibration, is found to be at 738 cm^-1^ according to the calculation.

Figure S 58. Exert of the experimental and calculated IR spectra of **1**. Level of theory: B3LYP, def2-TZV.

Figure S 59. TD-DFT calculated UV/Vis spectra of **1** and of potential decomposition products. Level of theory: B3LYP, def2-TZV.

Figure S 60. Comparison of the normalised experimental and TD-DFT calculated UV/Vis spectra of **1** in DCM **1** in DCM solution (5 · 10^-5^ mol L^-1^). Level of theory: B3LYP, def2-TZV.

Table S 9. Calculated electronic Tolman parameters for complexes of the type [Ni(CO)_3_PR_3_]. Level of theory: B3LYP, def2-TZV. ^F^stb = trans-1,2-bis(4-(trifluoromethyl)phenyl)ethene

| Ligand | A_1_ CO [cm^-1^] |
| --- | --- |
| PF_3_ | 2173 |
| ^F^stb | 2146* |
| P(C_6_F_5_)_3_ | 2144 |
| FPPh_2_ | 2141 |
| PPh_3_ | 2130 |
| P^t^Bu_3_ | 2120 |

* The TEP was calculated on the basis of [Ni(CO)_2_(^F^stb)], due to the high sterical demand of the stilbene ligand. Therefore, no direct comparison regarding the CO vibrational frequencies between the other complexes of the type [Ni(CO)_3_PR_3_] can be made. In the case of the [Ni(CO)_3_(^F^stb)] no symmetric A1 stretching frequency can be identified.

Table S 10. Major Loewdin reduced orbital populations per molecular orbital with respect to nickel. Level of theory: B3LYP, def2-TZVP. COD= cycloocta-1,5-diene, L = 2,3,4,5-tetraphenylthiophene-1-oxide, ^F^stb = trans-1,2-bis(4-(trifluoromethyl)phenyl)ethene

|  | **[Ni(FPPh_2_)_4_]** | **[Ni(COD)_2_]** | **[Ni(COD)L]** | **[Ni(^F^stb)_3_]** |
| --- | --- | --- | --- | --- |
| HOMO | 10 % Ni s orbital, 34 % Ni d orbital | 7 % Ni s orbital, 39 % Ni d orbital | 5 % Ni p orbital,16 % Ni d orbital | 4 % Ni s orbital, 65 % Ni d orbital |
| LUMO | 10 % Ni d orbital | 24 % Ni d orbital | 26 % Ni d orbital | No contributions at nickel centre |

Table S 11. Lowedin charges from Loewdin population analysis. Level of theory: B3LYP, def2-TZVP. L = 2,3,4,5-tetraphenylthiophene-1-oxide, ^F^stb = trans-1,2-bis(4-(trifluoromethyl)phenyl)ethene

|  | **[Ni(FPPh_2_)_4_]** | **[Ni(COD)_2_]** | **[Ni(cod)L]** | **[Ni(^F^stb)_3_]** |
| --- | --- | --- | --- | --- |
| Ni | -1.041795 | -0.267384 | -0.164029 | -0.083402 |

Table S 12. CHELPG charges. Level of theory: B3LYP, def2-TZVP. L = 2,3,4,5-tetraphenylthiophene-1-oxide, ^F^stb = trans-1,2-bis(4-(trifluoromethyl)phenyl)ethene

|  | **[Ni(FPPh_2_)_4_]** | **[Ni(COD)_2_]** | **[Ni(cod)L]** | **[Ni(^F^stb)_3_]** |
| --- | --- | --- | --- | --- |
| Ni | -0.269381 | 0.811235 | -0.121187 | 0.471720 |

Table S 13. Natural charges from natural population analysis. Level of theory: B3LYP, def2-TZVP. COD = cycloocta-1,5-diene, L = 2,3,4,5-tetraphenylthiophene-1-oxide, ^F^stb = trans-1,2-bis(4-(trifluoromethyl)phenyl)ethene

|  | **[Ni(FPPh_2_)_4_]** | **[Ni(COD)_2_]** | **[Ni(cod)L]** | **[Ni(^F^stb)_3_]** |
| --- | --- | --- | --- | --- |
| Ni | 0.00504 | 0.64169 | 0.71213 | 0.65822 |

Table S 14. Second order perturbation analyses for [Ni(PFPh_2_)_4_]. "LP" for 1-center valence lone pair and “LV” for lone vacant orbital. Threshold ≥ 5 kcal/mol. Level of theory: B3LYP, def2-TZVP.

| **Interactions** | **Stabilisation energy [kcal/mol]** | **Orbital description** |
| --- | --- | --- |
| Ni 🡪 X | | |
| Ni d orbital 🡪 P p* orbital | 6.5 | LP(4)Ni24 🡪 LV(1)P23 |
| Ni d orbital 🡪 P p* orbital | 6.5 | LP(4)Ni24 🡪 LV(1)P48 |
| Ni d orbital 🡪 P p* orbital | 6.5 | LP(4)Ni24 🡪 LV(1)P72 |
| Ni d orbital 🡪 P p* orbital | 6.5 | LP(4)Ni24 🡪 LV(1)P96 |
|  |  |  |
| X 🡪 Ni | | |
| P sp hybrid orbital 🡪 Ni s* orbital | 75.0 | LP(1)P23 🡪 LV(1)Ni24 |
| P sp hybrid orbital 🡪 Ni s* orbital | 75.0 | LP(1)P48 🡪 LV(1)Ni24 |
| P sp hybrid orbital 🡪 Ni s* orbital | 75.0 | LP(1)P72 🡪 LV(1)Ni24 |
| P sp hybrid orbital 🡪 Ni s* orbital | 75.0/nbo/ | LP(1)P96 🡪 LV(1)Ni24 |

Table S 15. Second order perturbation analyses for [Ni(COD)_2_]. "LP" for 1-center valence lone pair and “LV” for lone vacant orbital. Threshold ≥ 5 kcal/mol. Level of theory: B3LYP, def2-TZVP. COD = cycloocta-1,5-diene

| **Interactions** | **Stabilisation energy [kcal/mol]** | **Orbital description** |
| --- | --- | --- |
| Ni 🡪 X | | |
| Ni d orbital 🡪 p* orbital | 15.2 | LP(3)-Ni9 🡪 BD*(2)C4-C5 |
| Ni d orbital 🡪 p* orbital | 17.7 | LP(4)-Ni9 🡪 BD*(2)C1-C8 |
| Ni d orbital 🡪 p* orbital | 7.1 | LP(5)-Ni9 🡪 BD*(2)C4-C5 |
| Ni d orbital 🡪 p* orbital | 15.3 | LP(3)-Ni9 🡪 BD*(2)C13-C14 |
| Ni d orbital 🡪 p* orbital | 17.5 | LP(4)-Ni9 🡪 BD*(2)C10-C17 |
| Ni d orbital 🡪 p* orbital | 7.1 | LP(5)-Ni9 🡪 BD*(2)C10-C17 |
| X 🡪 Ni | | |
| C-C p orbital 🡪 Ni s* orbital | 15.8 | BD(2)C10-C17🡪 LV(1)Ni9 |
| C-C p orbital 🡪 Ni s* orbital | 15.7 | BD(2)C13-C14 🡪 LV(1)Ni9 |
| C-C p orbital 🡪 Ni s* orbital | 15.7 | BD(2)C1-C8 🡪 LV(1)Ni9 |
| C-C p orbital 🡪 Ni s* orbital | 15.8 | BD(2)C4-C5 🡪 LV(1)Ni9 |

Table S 16. Second order perturbation analyses for [Ni(COD)L]. "LP" for 1-center valence lone pair and “LV” for lone vacant orbital. Threshold ≥ 5 kcal/mol. Level of theory: B3LYP, def2-TZVP. COD = cycloocta-1,5-diene, L = 2,3,4,5-tetraphenylthiophene-1-oxide

| **Interactions** | **Stabilisation energy [kcal/mol]** | **Orbital description** |
| --- | --- | --- |
| Ni 🡪 X | | |
| Ni d orbital 🡪 C-C p* orbital | 14.8 | LP(5)Ni1 🡪 BD*(2)C24-C25 |
| Ni d orbital 🡪 C-C p* orbital | 15.5 | LP(5)Ni1 🡪 BD*(2)C26-C27 |
| Ni d orbital 🡪 C-C p* orbital | 13.9 | LP(3)Ni1 🡪 BD*(2)C14-C16 |
| Ni d orbital 🡪 C-C p* orbital | 7.9 | LP(3)Ni1 🡪 BD*(2)C4-C6 |
| Ni d orbital 🡪 C-C p* orbital | 15.9 | LP(4)Ni1 🡪 BD*(2)C4-C6 |
| Ni d orbital 🡪 C-C p* orbital | 7.3 | LP(4)Ni1 🡪 BD*(2)C14-C16 |
| X 🡪 Ni | | |
| S sp hybrid orbital 🡪 Ni s* orbital | 5.2 | LP(1)S2 🡪 LV(1)Ni1 |
| C-C p orbital 🡪 Ni s* orbital | 8.7 | BD(2)C24-C25 🡪 LV(1)Ni1 |
| C-C p orbital 🡪 Ni s* orbital | 7.6 | BD(2)C26-C27 🡪 LV(1)Ni1 |
| C-C p orbital 🡪 Ni s* orbital | 15.4 | BD(2)C4-C6 🡪 LV(1)Ni1 |
| C-C p orbital 🡪 Ni s* orbital | 16.1 | BD(2)C14-C16 🡪 LV(1)Ni1 |

Table S 17. Second order perturbation analyses for [Ni(^F^stb)]. "LP" for 1-center valence lone pair and “LV” for lone vacant orbital. Threshold ≥ 5 kcal/mol. Level of theory: B3LYP, def2-TZVP. ^F^stb = trans-1,2-bis(4-(trifluoromethyl)phenyl)ethene

| **Interactions** | **Stabilisation energy [kcal/mol]** | **Orbital description** |
| --- | --- | --- |
| Ni 🡪 X | | |
| Ni d orbital 🡪 C-C p* orbital | 10.4 | LP(4)Ni94 🡪 BD*(2)C1-C14 |
| Ni d orbital 🡪 C-C p* orbital | 18.0 | LP(5)Ni94 🡪 BD*(2)C1-C14 |
| Ni d orbital 🡪 C-C p* orbital | 23.9 | LP(5)Ni94 🡪 BD*(2)C27-C40 |
| Ni d orbital 🡪 C-C p* orbita | 27.0 | LP(4)Ni94 🡪 BD*(2)C53-C66 |
| X 🡪 Ni | | |
| C-C p orbital 🡪 Ni s orbital | 8.8 | BD(2)C1-C14 🡪 LV(1)Ni94 |
| C-C p orbital 🡪 Ni s orbital | 8.8 | BD(2)C27-C40 🡪 LV(1)Ni94 |
| C-C p orbital 🡪 Ni s orbital | 8.7 | BD(2)C53-C66 🡪 LV(1)Ni94 |

# Literature

[1] A. Sen, T.-W. Lai, R. R. Thomas, *J. Organomet. Chem.* **1988**, *358*, 567-588.

[2] J. P. Wolfe, S. L. Buchwald, *J. Am. Chem. Soc.* **1997**, *119*, 6054-6058.

[3] A. Manzoor, P. Wienefeld, M. C. Baird, P. H. M. Budzelaar, *Organometallics* **2017**, *36*, 3508-3519.

[4] L. Riesel, J. Haenel, G. Ohms, *J. Fluor. Chem.* **1988**, *38*, 335-340.

[5] in *DAISY, part of TopSpin 4.1.1*, Bruker BioSpin GmbH, Rheinstetten, **2020**.

[6] G. Mavel, *Ann. Rep. NMR Spectr.* **1973**, *5B*, 1.

[7] L. P. Miller, J. A. Vogel, S. Harel, J. M. Krussman, P. R. Melvin, *Org. Lett.* **2023**, *25*, 1834-1838.

[8] Stoe&Cie, in *Darmstadt, Germany, Vol. X-RED, Program for data reduction and absorption correction*, Program for data reduction and absorption correction 1.28b ed., Stoe & Cie GmbH, Darmstadt, Germany, **2005**, pp. X-RED, Program for data reduction and absorption correction.

[9] G. M. Sheldrick, *Acta. Cryst. A* **2015**, *71*, 3-8.

[10] G. M. Sheldrick, *Acta. Cryst. B* **2015**, *71*, 3-8.

[11] L. J. Farrugia, *J. Appl. Crystallogr.* **2012**, *45*, 849-854.

[12] K. Brandenburg, M. Berndt, in *Diamond*, 4.6.8 ed., Crystal Impact, Bonn, Germany, **1999**, pp. Diamond - Crystal and Molecular Structure Visualization.

[13] A. F. Gushwa, Y. Belabassi, J.-L. Montchamp, A. F. Richards, *J. Chem. Crystallogr.* **2008**, *39*, 337-347.

[14] F. L. Hirshfeld, *Acta. Cryst. A* **1976**, *A32*, 239-244.

[15] a) A. M. A. Boshaala, S. J. Simpson, J. Autschbach, S. Zheng, *Inorg. Chem.* **2008**, *47*, 9279-9292; b) Y. Morino, K. Kuchitsu, T. Moritani, *Inorg. Chem.* **1969**, *8*, 867-871.

[16] a) H. Oberhammer, R. Schmutzler, O. Stelzer, *Inorg. Chem.* **1978**, *17*, 1254-1258; b) M. Fild, R. Schmutzler, *J. Chem. Soc. A.* **1970**, 2359-2364.

[17] N. Fey, M. Garland, J. P. Hopewell, C. L. McMullin, S. Mastroianni, A. G. Orpen, P. G. Pringle, *Angew. Chem., Int. Ed.* **2012**, *51*, 118-122.

[18] L. Riesel, J. Haenel, *Z. Anorg. Allg. Chem.* **1991**, *603*, 145-150.

[19] L. Riesel, D. Sturm, A. Nagel, S. Taudien, A. Beuster, A. Karwatzki, *Z. Anorg. Allg. Chem.* **1986**, *542*, 157-166.

[20] A. B. Burg, *Inorg. Nucl. Chem. Letters* **1977**, *13*, 199-203.

[21] Tsutomu Mizuta, T. Yamasaki, H. Nakazawa, K. Miyoshi, *Organometallics* **1996**, *15*, 1093-1100.

[22] D. Gudat, A. Haghverdi, H. Hupfer, M. Nieger, *Chem. Eur. J.* **2000**, *6*, 3414-3425.

[23] P. M. Miura-Akagi, T. W. Chapp, W. Y. Yoshida, G. P. A. Yap, A. L. Rheingold, R. P. Hughes, M. F. Cain, *Organometallics* **2023**, *42*, 672-688.

[24] A. B. Burg, G. B. Street, *Inorg. Chem.* **1966**, *5*, 1532-1537.

[25] W. S. Sheldrick, O. Stelzer, *J. Chem. Soc., Dalton Trans.* **1973**, 926-929.

[26] M. W. Bezpalko, B. M. Foxman, C. M. Thomas, *Inorg. Chem.* **2015**, *54*, 8717-8726.

[27] a) S. A. Macgregor, D. C. Roe, W. J. Marshall, K. M. Bloch, V. I. Bakhmutov, V. V. Grushin, *J. Am. Chem. Soc.* **2005**, *127*, 15304-15321; b) V. V. Grushin, W. J. Marshall, *J. Am. Chem. Soc.* **2004**, *126*, 3068-3069.

[28] F. C. Bradley, E. H. Wong, *Inorg. Chim. Acta* **1986**, *120*, L21-L22.

[29] R. H. Morris, J. F. Sawyer, C. T. Schweitzer, A. Sella, *Organometallics* **1989**, *8*, 2099-2106.

[30] D. R. Anton, R. H. Crabtree, *Organometallics* **1983**, *2*, 855-859.

[31] D. Gärtner, S. Sandl, A. J. v. Wangelin, *Catal. Sci. Technol.* **2020**, *10*, 3502-3514.

[32] S. Baweja, T. Gabler, P. Lönnecke, E. Hey-Hawkins, *Dalton Trans.* **2023**, *52*, 6494-6500.

[33] a) T. M. Maier, S. Sandl, P. Melzl, J. Zweck, A. J. v. Wangelin, R. Wolf, *Chem. Eur. J.* **2020**, *26*, 6113-6117; b) J. A. Widegren, R. G. Finke, *J. Mol. Catal. A Chem.* **2003**, *198*, 317-341.

[34] a) F. Neese, *WIREs Comput. Mol. Sci.* **2017**, *8*, e1327; b) F. Neese, *WIREs Comput. Mol. Sci.* **2011**, *2*, 73-78.

[35] a) S. Grimme, J. Antony, S. Ehrlich, H. Krieg, *J. Phys. Chem.* **2010**, *132*, 154104; b) S. Grimme, S. Ehrlich, L. Goerigk, *J. Comp. Chem.* **2011**, *32*, 1456-1465.

[36] F. Neese, F. Wennmohs, A. Hansen, U. Becker, *Chem. Phys.* **2009**, *356*, 98-109.

[37] a) A. D. Becke, *J. Phys. Chem.* **1993**, *98*, 5648-5652; b) P. J. Stephens, F. J. Devlin, C. F. Chabalowski, M. J. Frisch, *J. Chem. Phys.* **1994**, *98*, 11623-11627.

[38] a) F. Weigend, *Phys. Chem. Chem. Phys.* **2006**, *8*, 1057-1065; b) F. Weigend, R. Ahlrichs, *Phys. Chem. Chem. Phys.* **2005**, *7*, 3297-3305.

[39] a) J. Tao, J. P. Perdew, V. N. Staroverov, G. E. Scuseria, *Phys. Rev. Lett.* **2003**, *91*, 146401-146404; b) V. N. Staroverov, G. E. Scuseria, J. Tao, J. P. Perdew, *J. Phys. Chem.* **2004**, *121*, 11507.

[40] C. M. Breneman, K. B. Wiberg, *J. Comp. Chem.* **1990**, *11*, 361-373.

[41] a) A. E. Reed, L. A. Curtiss, F. Weinhold, *Chem. Rev.* **1988**, *88*, 899-926; b) A. E. Reed, R. B. Weinstock, F. Weinhold, *J. Phys. Chem.* **1985**, *83*, 735-746.

[42] E. D. Glendening, K. B. J, A. E. Reed, J. E. Carpenter, J. A. Bohmann, C. M. Morales, P. Karafiloglou, C. R. Landis, F. Weinhold, Theoretical Chemistry Institute, University of Wisconsin, Madison, **2018**, p. NBO 7.0.

[43] S. Hanf, A. L. Colebatch, P. Stehr, R. García-Rodríguez, E. Hey-Hawkins, D. S. Wright, *Dalton Trans.* **2020**, *49*, 5312-5322.

[44] C. Adamo, D. Jacquemin, *Chem. Soc. Rev.* **2013**, *42*, 845-856.
